# Immersion‐Based Clearing and Autofluorescence Quenching in Myocardial Tissue

**DOI:** 10.1111/micc.70034

**Published:** 2025-11-05

**Authors:** Victoria E. Sturgess, Nadia K. Korovesis, Domingo E. Uceda, Ali Citalan‐Madrid, Eric V. Vu, Katherine Stangis, Françoise Van den Bergh, Salman I. Essajee, Binyamin Jacobovitz, Gregory B. Sands, Daniel A. Lawrence, Johnathan D. Tune, Daniel A. Beard, Geoffrey G. Murphy, C. Alberto Figueroa

**Affiliations:** ^1^ Department of Biomedical Engineering University of Michigan Ann Arbor Michigan USA; ^2^ Section of Vascular Surgery, Department of Surgery University of Michigan Ann Arbor Michigan USA; ^3^ Michigan Neuroscience Institute University of Michigan Medical School Ann Arbor Michigan USA; ^4^ Department of Molecular and Integrative Physiology University of Michigan Ann Arbor Michigan USA; ^5^ College of Literature, Science, and the Arts University of Michigan Ann Arbor Michigan USA; ^6^ Department of Physiology and Anatomy University of North Texas Health Science Center Fort Worth Texas USA; ^7^ Microscopy Core, Biomedical Research Core Facilities University of Michigan Ann Arbor Michigan USA; ^8^ Auckland Bioengineering Institute University of Auckland Auckland New Zealand; ^9^ Department of Internal Medicine University of Michigan Medical School Ann Arbor Michigan USA

**Keywords:** confocal microscopy, CUBIC, delipidation, imaging depth, myocardial microvasculature

## Abstract

**Objective:**

Recent innovations in optical microscopy and tissue preparation permit 3D visualization of complex microvascular networks. Tissue clearing techniques improve light penetration and extend imaging depth. Typically, perfusion‐based approaches are used for vascular labeling and tissue clearing. However, immersion‐based methodologies provide enhanced practicality when processing tissues from larger animal models.

**Methods:**

We present an immersion‐based microvascular labeling and tissue clearing protocol for myocardial tissues using tomato lectin and CUBIC (Clear, Unobstructed Brain/Body Imaging Cocktails and Computational analysis), demonstrating success for imaging depths of up to 150 μm. This protocol optimized the delipidation and quenching stages for rat and pig myocardial tissues. Image quality was assessed using an automated analysis of signal‐to‐noise ratios (SNR) and average z‐slice intensities.

**Results:**

Optimal image quality was obtained with 24‐h CUBIC Reagent I incubation times. Quenching agents TrueVIEW, Glycine, and Trypan Blue did not significantly impact SNR values. TrueBlack and Sudan Black B showed trends of reduced imaging depth compared to controls without quencher incubation. Overall, rat myocardial tissues had higher SNRs than pig tissue samples.

**Conclusions:**

This protocol provides a rigorous foundation for the optimization of immersion‐based approaches for myocardial tissue clearing. Future studies will quantify anatomical and topological coronary microvascular to compare disease states.

**Abbreviations**
ANOVA: Analysis of Variance
BSA: Bovine Serum Albumin
CUBIC: Clear, Unobstructed Brain/Body Imaging Cocktails and Computational analysis
DI water: De‐ionized water
FITC Dextran: fluorescein isothiocyanate conjugated dextran
LEL:
*Lycopersicon esculentum* lectin
PBS: phosphate‐buffered saline
PFA: paraformaldehyde
−Q/+TL: sample without quencher incubation step but with tomato lectin incubation step
−Q/−TL: sample without quencher incubation step and without tomato lectin incubation step
SNR: Signal to Noise Ratio
TB: TrueBlack (used in figures)
Tukey HSD: Tukey honest significance tests
w/v: weight‐per‐volume
WGA: wheat germ agglutinin

## 1 Introduction

The microvasculature is essential for the exchange of biomolecules and gases in tissues. Specifically, myocardial microvasculature supports the critical demand for oxygen and nutrients in cardiomyocytes. Cardiac diseases are associated with structural and functional alterations in myocardial microvascular networks. For instance, reductions in microvascular density have been reported in patients with heart failure with preserved ejection fraction [1] and significant microvascular remodeling has been shown in myocardial tissue of spontaneously hypertensive rats [2]. Visualization of microvascular networks of small arterioles, small venules, and capillaries, particularly in 3D, can help describe microvascular structural adaptations in disease states and understand their impact on myocardial perfusion [2, 3, 4].

### 1.1 Microvascular Imaging Methods

Microvascular imaging is a dynamic field. A wide range of methods have been deployed to optimize the visualization of microvessels in different tissues. In 1661, Marcello Malpighi was the first to demonstrate the existence of capillaries using an early microscope [5]. Subsequently, 2D histology has been used in the field of vascular pathology to describe vessel wall composition and to quantify vascular density [1, 6]. However, histology data are limited to thin slices and do not provide information on the 3D connectivity of microvascular networks. Alternatively, 3D volumetric data of labeled vasculature can be acquired with imaging modalities such as micro‐computed tomography (micro‐CT) or fluorescence microscopy of optically clear tissue. Labels in micro‐CT include dyes (in vivo) and casting materials (ex vivo) that help delineate the boundary between vessels and surrounding tissues. Complex arterial and venous vasculature can be visualized with micro‐CT [7, 8]. However, micro‐CT applications to capillary networks have been hindered due to limited spatial resolutions and incomplete capillary filling due to the viscosity of the casting materials [9]. Sub‐micron spatial resolution can be obtained with fluorescent imaging modalities such as confocal microscopy. Vessels can be fluorescently labeled with dyes or gelatin‐based casts and visualized in relation to other tissue structures [10, 11, 12].

### 1.2 Vascular Perfusion

Vascular perfusion is commonly used for passing dyes or vessel‐filling materials through vascular networks. For example, elastomer casting methods include perfusing vasculature with a silicone elastomer and using methyl salicylate to render the surrounding tissue transparent [13]. Bassingthwaighte et al. [14] and Kassab and Fung [15] used this approach to visualize capillary connectivity within the myocardium. Similar vascular perfusion can be used in ex vivo micro‐CT and fluorescence microscopy with vessel‐filling agents such as radiopaque silicon rubber and fluorescently labeled gelatin mixtures, respectively. Perfusion pressure, material viscosity, and capillary leaking need to be considered when working with vascular filling methods [9]. In fluorescence microscopy, vascular stains can also be applied with in vivo or ex vivo perfusion [16, 17, 18]. Perfusion‐based methods are typically preferred when working with vascular endothelial stains because they provide a more direct application of the stain, and imaging depth is not limited by diffusion. While perfusion‐based methods are useful for whole‐organ labeling, they require the cannulation of a large vessel. These methods are not suitable when only small sections or biopsies of tissue are available, limiting their use in large‐animal studies or with donor tissue samples.

### 1.3 Immersion‐Based Methods

Instead of perfusion, immersion‐based methods rely on the diffusion of labels through the tissue. In fluorescence microscopy, immersion methods have been used to label non‐vascular structures such as myocytes and fibroblasts [19, 20]. Previous work has demonstrated that diffusion of antibodies and lectins can be used as vascular labels in myocardial samples at tissue depths of up to 500 μm [21, 22]. Immersion‐based techniques are an appealing alternative when working with small tissue samples, including donor tissue samples.

### 1.4 Tissue Clearing for Fluorescence Microscopy

In fluorescence microscopy, imaging depth is hindered by light scattering and light‐absorbing properties of biological tissues. Tissue‐clearing methods are used to correct refractive index (RI) mismatches in highly heterogeneous tissues and to reduce the light scattering that occurs due to this mismatch [23, 24]. Techniques include removal of lipids and other light‐scattering molecules, dissociation of collagen, or dehydration of samples. Additionally, de‐colorization, or the removal of light‐absorbing pigments, allows light to penetrate deeper into tissues [24]. The CUBIC (Clear, Unobstructed Brain Imaging Cocktails and Computational Analysis) protocol was designed for whole‐brain imaging and has subsequently been used to clear other organs and tissues [25, 26, 27, 28]. For heme‐rich organs such as the myocardium, CUBIC has often been used with a vascular perfusion approach [26]. Whole‐heart perfusion‐based clearing has allowed for 3D reconstruction of capillary networks within approximately 0.6 mm thick slices along the short axis of the myocardium [29]. Similar to vascular labeling, perfusion‐based techniques are suitable for tissue clearing when the whole heart is available but are not feasible when working with small tissue sections. Immersion‐based approaches to tissue clearing are required instead. It is worth noting that immersion‐based methods of other clearing reagents, including uDISCO, CLARITY, and SHIELD, have been tested in myocardial tissues for imaging muscle fiber direction with Wheat‐Germ Agglutinin (WGA) [30].

### 1.5 Autofluorescence Quenching

Tissue autofluorescence is another important consideration in fluorescent confocal microscopy. Myocardial tissues contain high levels of both heme and lipofuscin, two strong autofluorescent pigments. Additionally, the process of paraformaldehyde (PFA) fixation can lead to fluorescent crosslinking, adding more background noise to an already autofluorescent tissue [31]. To address these challenges, different autofluorescence quenching agents have been identified. Zhang et al. investigated the performance of various autofluorescence quenchers in fixed myocardial tissue to enhance signal‐to‐noise ratios (SNR) of 2D, *non‐cleared*, myocardium samples [32]. However, to our knowledge, few studies have evaluated the impact of quenching on imaging depth in any tissue type [33, 34] and no studies so far have investigated the compatibility of autofluorescence quenching agents with *cleared* myocardial tissues.

In this study, we outline an immersion‐based protocol for tissue clearing and vascular labeling and apply it to myocardial tissue from both small (rat) and large (pig) mammals. This protocol uses Sca*l*eCUBIC (CUBIC) tissue‐clearing reagents and tomato lectin vascular labeling to image 300‐μm sections from the left ventricular free wall with confocal microscopy. We optimize the tissue‐clearing steps of the protocol by analyzing SNR and imaging depth. Further, we incorporate an optional autofluorescence quenching step, evaluating the impact of various quenching agents on tissue clarity, to explore potential tradeoffs between improved SNR and hindered image depth.

Our results show that the complete immersion‐based protocol is successful for imaging microvascular structures up to 150 μm deep within tissue. Additionally, results suggest that moderate CUBIC Reagent I incubation times of 12 or 24 h provide optimal SNR for this protocol. Furthermore, although the use of lipofuscin quenching dyes such as TrueBlack and Sudan Black B generally improves SNR at the tissue surface, they also diminish overall imaging depth. Other quenchers, such as TrueVIEW and Glycine, showed potential for improved SNR and imaging depth and are worth further consideration. To our knowledge, this is the first paper to successfully image myocardial microvascular networks at the capillary level using a completely immersion‐based protocol and to show how SNR decays with imaging depth. We believe this work will provide a helpful foundation for 3D imaging of microvascular networks in myocardial tissue sections, a key step towards morphometric quantification and biophysical simulation.

## 2 Materials and Methods

### 2.1 Tissue Collection

This study used myocardial tissue from rats and pigs that were supplied by collaborating labs at the University of Michigan and the University of North Texas Health Science Center, respectively. All the protocols involving animals conformed to the National Institutes of Health Guide for the Care and Use of Laboratory Animals and were approved by the University of Michigan Animal Research Committee or the University of North Texas Health Science Center Institutional Animal Care and Use Committee.

Through collaborating laboratories, we acquired both murine and swine heart specimens to minimize animal use. Four murine hearts from healthy male Sprague–Dawley rats (Beard Lab, University of Michigan) were harvested under anesthesia and perfused with PBS. Additionally, a swine heart from a 4‐year‐old male Ossabaw pig that underwent a ventricular pacing protocol [35] (Tune Lab, University of North Texas Health Science Center) was harvested following electrically induced cardiac fibrillation. Transmural myocardial tissue sections from the anterior left ventricular free wall were shipped overnight to the University of Michigan in cardioplegia with protease inhibitors.

### 2.2 Tissue Preparation

Unless otherwise labeled, all reagents were obtained from Fisher Scientific, USA. Pig tissues were washed twice in PBS for 1 h after removal from cardioplegia and prior to fixation. All PBS wash steps in this protocol were performed with 1X PBS at 22°C. Rat and pig tissues were fixed using a 4% PFA solution at 4°C for 24–48 h. After fixation, myocardial samples were mounted in 4% agarose and sectioned on a vibratome at 100 μm thickness for immunofluorescence and 300 μm thickness for tissue clearing experiments. All samples were stored in PBS at 4°C prior to further labeling or clearing steps and used within 1 month.

### 2.3 Vascular Labeling Reagents

*L. esculentum* tomato lectin Dylight 594 [Invitrogen] was made by diluting the stock sample in PBS for a 10 μg/mL concentration. *L. esculentum* tomato lectin will be referred to as tomato lectin or LEL. CD31 labeling reagents dilutions were made with blocking‐permeabilization buffer consisting of 3% w/v BSA, 0.6% v/v Triton X‐100 [Sigma‐Aldrich], and 0.05% w/v sodium azide [Fluka] in PBS. The primary and secondary antibodies are a 1:200 dilution of rabbit anti‐CD31 [Bioss, catalog # BS‐0468R] and a 1:500 dilution of donkey anti‐rabbit [Invitrogen, catalog # A‐21206].

### 2.4 Tomato Lectin and CD31 Colocalization

100 μm tissue sections were incubated at 37°C overnight in the permeabilization‐blocking buffer. Subsequently, tissues were incubated in the primary CD31 antibody solution for 24 h at 4°C and washed in PBS. Samples were then incubated in a secondary antibody solution with tomato lectin at 4°C, washed with PBS, and mounted in mineral oil for imaging.

### 2.5 Tissue Clearing Reagents

Sca*l*eCUBIC‐1 (Reagent I) and Sca*l*eCUBIC‐2 (Reagent II) were prepared according to Suzaki et al. [26]. Reagent II had a measured refractive index of 1.49. Of note, we elected to omit the optional Triton X‐100 from our Reagent II mixture, as the manufacturer of TrueBlack reports it to be intolerant of detergents. Dilutions of Reagent I and II were made with deionized water and PBS, respectively.

### 2.6 Quenching Reagents

Five quenching agents were used (Table 1). TrueBlack and TrueVIEW were prepared according to manufacturers' instructions. Sudan Black B was diluted to 0.1% (W/V) in 70% ethanol [36]. Glycine was diluted to 0.3M in PBS, and Trypan Blue was diluted to 0.05% (W/V) in PBS [32, 36]. Additionally, a 1X quencher wash buffer was made of 137 mM NaCl, 2.6 mM KCl, 10 mM Na2HPO4, 1.76 mM KH2PO4, 0.005% sodium azide [Fluka Biochemika], corrected to pH 8.0, and stored at 4°C.

**TABLE 1:** Quenching agent preparation and information.

Quenching agent	Preparation	Incubation duration	Targeted autofluorescent source	Vendor information
TrueBlack	According to manufacturer instructions	1 min (airtight container)	Lipofuscin [36]	Cell Signaling Technologies
TrueVIEW	According to manufacturer instructions	5 min	Non‐Lipofuscin Sources (i.e., erythrocytes, collagen, and elastin) [37]	Vector Laboratories
Glycine	0.3 M, diluted in PBS	20 min	Formaldehyde Residues [38]	Fisher Scientific
Sudan Black B	0.1%, diluted in 70% ethanol	20 min (airtight container)	Lipofuscin [36, 39]	Sigma‐Aldrich
Trypan Blue	0.05%, diluted in PBS	15 min	Flavin Mononucleotide [40]	Sigma‐Aldrich

### 2.7 General Tissue Clearing and Autofluorescence Quenching Pipeline

**FIGURE 1:**
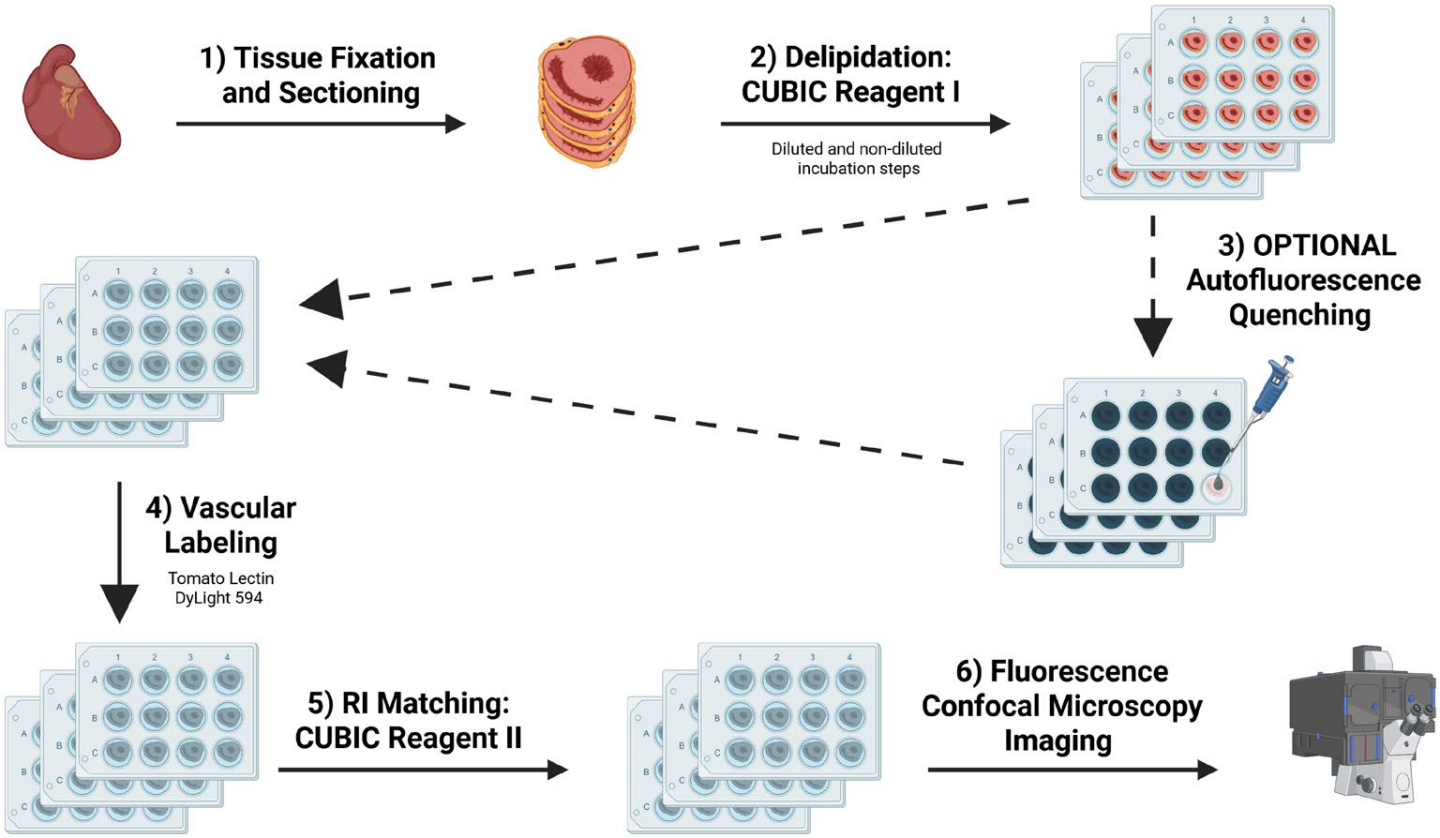
Overview of tissue clearing and autofluorescence quenching pipeline. Created with BioRender.com/0d0jti2.

The tissue clearing process is detailed in Figure 1.

1. Fixation and Sectioning. 300 μm‐thick sections were obtained for all tissue clearing experiments.
2. Tissue Delipidation with CUBIC Reagent I is split evenly into two phases: diluted and non‐diluted. Total incubation time for Reagent I is defined as the combined time spent in the two phases. Diluted concentration of Reagent I and total Reagent I incubation time were optimized (see Section 3.2). All incubation steps took place in a shaking incubator at 37°C. Following Reagent I incubation, the tissue was washed using PBS.
3. Optional Autofluorescence Quenching optimization was performed using one of five quenching agents (Table 1). When using quenching agents, a subsequent wash step was performed with a quencher wash buffer prior to step 4.
4. Sections were labeled by submersing tissue in tomato lectin. Submersed tissues were placed on a shaking incubator at 37°C for 24 h. Samples were wrapped in aluminum foil to block light and prevent photobleaching. Tissue wash was then performed using PBS.
5. Lastly, RI Matching was performed using CUBIC Reagent II. Samples were incubated in 50% Reagent II for 24 h, followed by 100% Reagent II for 48 h. All CUBIC Reagent II was performed at 22°C.
6. Tissues were mounted and prepared for microscopy. See Section 2.12

### 2.8 Delipidation Experiment

Two parameters of the Tissue Delipidation step were optimized: the concentration of Reagent I in the dilution phase and the total Reagent I incubation time. Diluted Reagent I concentrations were: 25% (ratio of 1 part Reagent I to 3 parts DI water), 50%, or 75%. Total Reagent I incubation times were split evenly between the diluted and undiluted phases and were: 2, 12, 24, 48, and 144 h. An additional sample with no Reagent I was also included as a control. This resulted in 16 experimental conditions, all performed without autofluorescence quenchers.

### 2.9 TrueBlack Experiment

A thorough analysis of the impact of TrueBlack quenching was performed by repeating the steps of the Delipidation Experiment with varying TrueBlack application times (30, 60, 300, and 600 s), producing a total of 64 different experimental conditions.

### 2.10 Comparative Autofluorescence Quenching Experiment

Further experiments were carried out to compare the performance of TrueBlack against the following autofluorescence quenchers: Sudan Black B, Glycine, TrueVIEW, and Trypan Blue. Diluted Reagent I concentration and total Reagent I incubation times were held constant for rat and pig tissues, with 12 ‐h incubation in 50% Reagent I followed by 12 h in 100% Reagent I (total Reagent I incubation time of 24 h). Based on results from the *TrueBlack Experiment*, incubation times were set to 60 s, while incubation times for the remaining quenching reagents were set according to literature values (Table 1) [32, 36]. Samples treated with autofluorescence quenchers were compared to two controls of unquenched samples without and with tomato lectin labeling. The first control provided a baseline of native tissue autofluorescence. The second control was used to assess the effectiveness of the quenchers in improving image quality. To account for experimental variability, two intra‐animal replicates (different sections of tissue from the same animal) were performed for each experimental condition. This resulted in a total of 7 experimental conditions (5 quenchers and the 2 controls above) and 14 samples.

### 2.11 Fluorescence Confocal Microscopy Imaging and Image Processing

Microscopy images were acquired on a Nikon A1 Confocal microscope using a 20× multi‐immersion Nikon A1plus lens. The refractive index of the objective was set at 1.52 (oil immersion setting) to match the refractive index of CUBIC Reagent 2 (~1.49). The objective had a numerical aperture of 0.75 and a working distance of 250–300 μm. For all images, an excitation wavelength of 561 nm was used, resulting in a lateral resolution of 456 nm and an axial resolution of 3032 nm [41].

Three‐dimensional volume reconstruction was achieved through sequential acquisition of two‐dimensional optical sections (1024 × 1024 pixels, lateral x, y sampling of 0.4 μm/pixel) at defined intervals along the *z*‐axis, collectively forming a z‐stack. Two different z‐step samplings were considered: (i) A coarse interval (5 μm) for assessment of tissue clearing quality and imaging depth; and (ii) A fine interval (0.975 μm) for vascular reconstruction. The coarse sampling resulted in 51 z‐slices for a total imaging depth of 250 μm. The higher‐resolution sampling provided volumetric imaging satisfying the Nyquist criteria (*z* interval smaller than axial resolution) and with a total imaging depth of 150 μm (155 slices) for a pig sample and 201.875 μm (207 slices) for a rat sample. Image acquisition and processing were performed using NIS‐Elements Advanced Research software (Nikon Instruments Inc.).

In the Tomato Lectin and CD31 Colocalization experiment, an image was collected using two wavelength channels (excitation wavelength of 488 and 516 nm) at a single z‐step location near the top of the sample. Binary images for each channel were constructed using the Niblack local thresholding method from the Python scikit‐image package [42, 43]. A window size of 55 × 55 pixels was used and the value for the local threshold is defined based on the mean (*μ*_*w*_) and standard deviation (*σ*_*w*_) of the window (Equation 1).

(1) $${\text{Threshold}}_{w}=\mu_{w}-\sigma_{w}$$

### 2.12 Quantitative Image Analysis

For each z‐stack image, quantitative analyses were performed using the Python packages ND2Reader [44] and Dask [45]. The analysis volume was defined as follows. First, z‐slice images were cropped in the x‐y plane to 512 × 512 pixel regions centered in the image. Due to surface roughness from tissue sectioning on the vibratome, the top of the sample was determined manually as the first z‐slice with vasculature covering the entire 512 × 512 pixel region. A total of 31 z‐slices, covering a depth of 150 mm, were included in the analysis.

Within a sample, the average intensity of the cropped region was calculated for each z‐slice. For each sample, a percent relative intensity was calculated as the ratio of the deepest average intensity (imaging depth of 150 μm) compared to the surface average intensity (imaging depth of 0 μm) and was reported as a percentage. In the TrueBlack experiments, some samples did not have the complete 150 μm imaging depth due to surface roughness. For these experiments, the average intensity for the deepest imaging depth consistent across all samples (105 μm) was used as the numerator.

(2) $$\text{Percent Relative Intensity}=\frac{\text{Average Intensity}\;\mathrm{at}\;150\;\left(\text{or}\;105\right)\;\mathrm{\mu m}}{\text{Average Intensity}\;\mathrm{at}\;0\;\mathrm{\mu m}}\times 100\%$$

**FIGURE 2:**
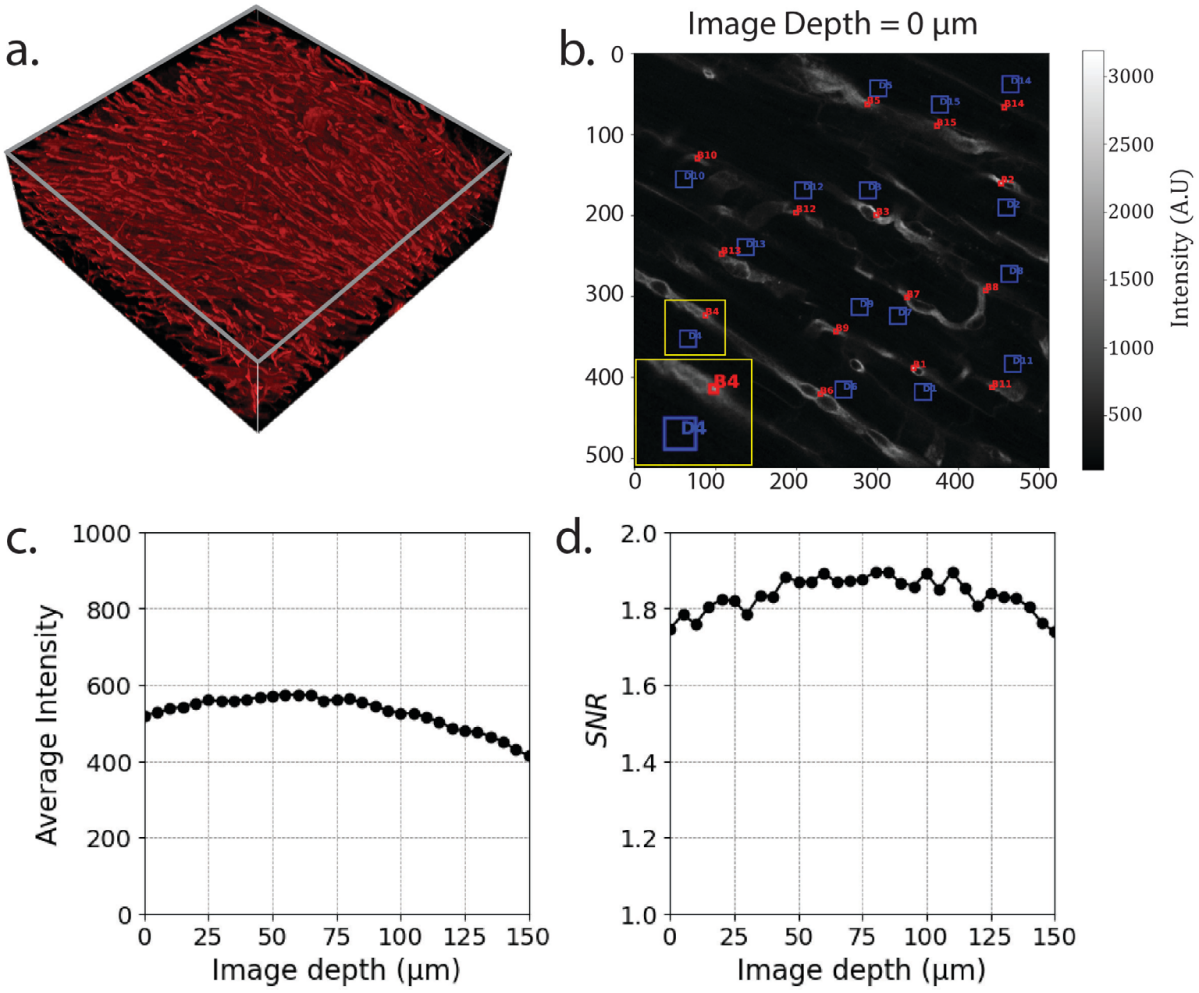
Quantitative image analysis workflow for a z‐stack (a). Bright vessel regions and dark tissue regions were identified (b) and used to calculate SNR for each slice in the z‐stack. Average slice intensity and SNR are plotted as a function of imaging depth (c, d).

Furthermore, an automated SNR analysis method was performed for each z‐slice. In each z‐slice, 15 regions with the highest average signal intensity, corresponding to vasculature structures, were identified using a 5 × 5 pixel sliding window. We refer to these as “bright regions” (see red boxes in Figure 2). The first bright region was identified as the brightest 5 × 5 pixel region. To avoid concentrating bright regions in small parts of the image, and to ensure sampling across different vessels within the image, a minimum distance between bright regions of 75 pixels was enforced. The first bright region was identified as the brightest 5 × 5 pixel region. Additional bright regions were identified as being the next brightest region that was at least 75 pixels away from all previously identified regions. For each bright region, a neighboring “dark region,” representative of the extravascular space, was identified. The dark region was selected as the area with the lowest standard deviation in signal intensity using a 20 × 20 pixel sliding window, constrained to be within a 61 × 61‐pixel search box centered around the bright region. For each z‐slice, 15 pairs of bright and dark regions were identified (Figure 2B). A local SNR value was calculated for each bright‐dark region pair (i) as the logarithm of the intensity mean (μ) in the bright region divided by the standard deviation of the intensity (σ) in the dark region (Equation 3). The mean of the 15 bright‐dark region SNR_i_ is then reported as the SNR of the z‐slice.

(3) $${\mathrm{SNR}}_{i}=\mathrm{lo}g_{10}{\left(\frac{\mu_{\text{bright region}}}{\sigma_{\text{dark region}}}\right)}_{i};\;\mathrm{SNR}=\text{mean}\;\left({\mathrm{SNR}}_{i}\right)$$

In the Tomato Lectin and CD31 colocalization experiment, separate SNR values were calculated for tomato lectin in the red channel (561 nm excitation wavelength) and CD31 in the green channel (488 nm excitation wavelength). Using binary images from the Niblack local thresholding, a corresponding DICE score was computed:

(4) $$\text{DICE score}=\frac{2\cdot \text{number of pixels in common in Tomato Lectin and}\;\mathrm{CD}31}{\text{number of pixels in Tomato Lectin}+\text{number of pixels in}\;\mathrm{CD}31}$$

### 2.13 Statistical Analysis

Two forms of variance exist within the data. Intra‐sample variance refers to the spread of the 15 SNR_i_ measurements across each z‐slice of one image. Inter‐sample variance refers to the spread of SNR measurements across a group of images at a single z‐slice position and accounts for variation among tissue samples. Statistical analyses within this study used inter‐sample variance. For the delipidation experiments, a two‐way analysis of variance (ANOVA) without interaction was performed to determine the effect of total Reagent I incubation time and diluted Reagent I concentration on percent relative intensity. Two‐way ANOVAs were also used to determine the effect of both variables on SNR, with separate analyses performed for imaging depths of 0 and 150 μm. For TrueBlack experiments, samples treated with CUBIC Reagent I were grouped based on TrueBlack incubation time. One‐way ANOVAs were performed to determine the effect of TrueBlack incubation time on percent relative intensity and SNR values at imaging depths of 0 and 105 μm. For the Comparative Autofluorescence Quenching Experiment, duplicates were run for each experimental condition. One‐way ANOVAs were performed to compare the effect of quencher type on percent relative intensity and SNR values at imaging depths of 0 and 150 μm. This analysis only included samples that received the tomato lectin label: the −Q/+ TL baseline and the 5 quencher types. For all ANOVAs, additional pairwise comparisons were performed using a Tukey Honest Significant Difference Test (Tukey HSD). Significance was defined as a *p* < 0.05 for all analyses.

### 2.14 Segmentation, Skeletonization and Morphometrical Quantification

For high‐resolution images, 3D reconstruction of microvascular networks was achieved through manual segmentation in the 3D Slicer software [46, 47, 48]. Skeletonization, diameter quantification, and graph construction were performed according to Vigneshwaran et al. [29] with minor modifications to the detection of vascular walls.

## 3 Results

### 3.1 Tomato Lectin and CD31 Colocalization

**FIGURE 3:**
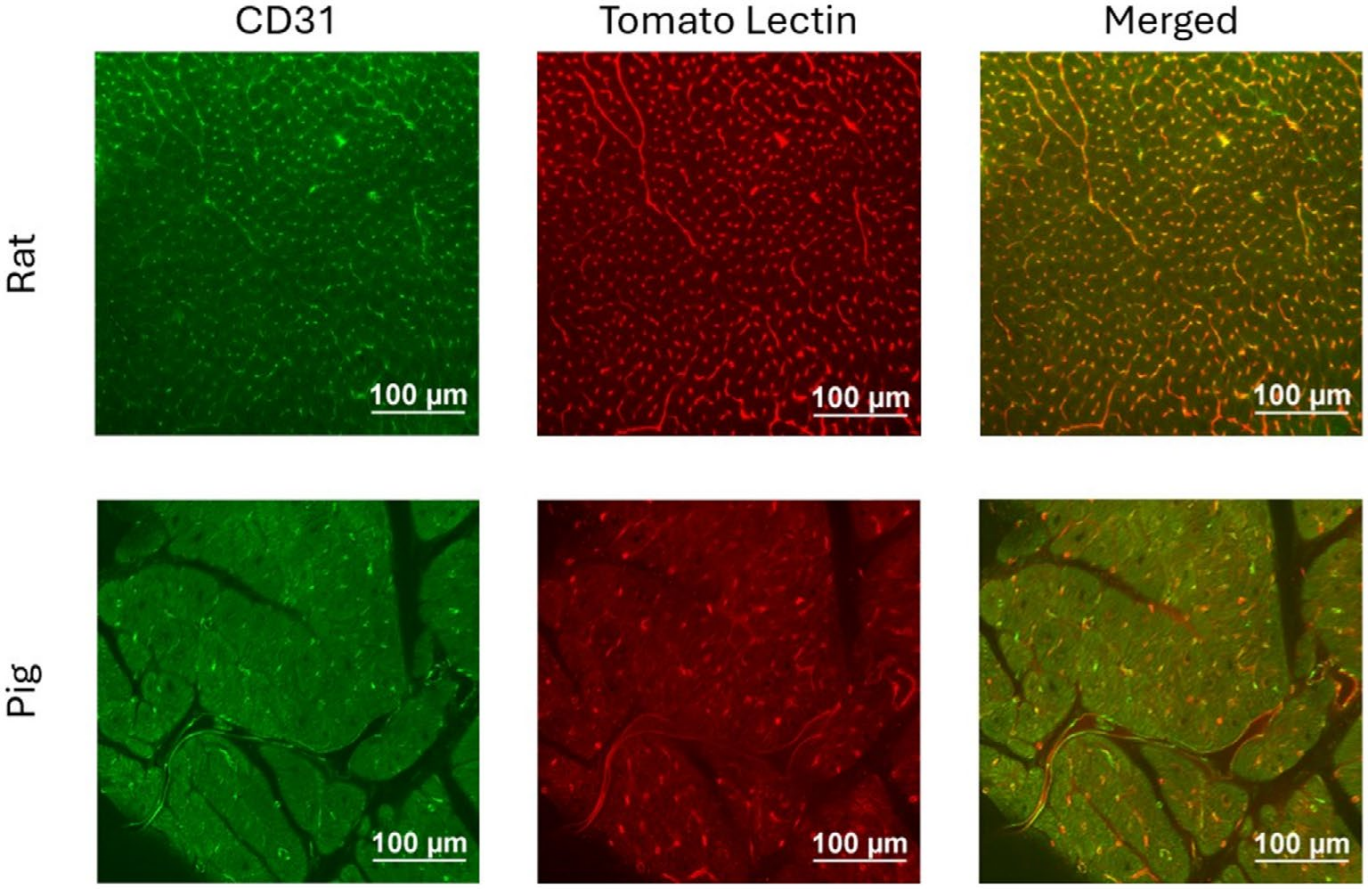
CD31 and tomato lectin colocalization in rat (top) and pig (bottom) samples. Left: CD31 stain in green. Middle: Tomato lectin in red. Right: Merged channels with colocalization appearing yellow.

Thin sections of rat and pig myocardium were stained using both CD31 immunofluorescence and tomato lectin (Figure 3). Results showed an overlap of fluorescence signals around vessel‐like structures. Tomato lectin appeared to stain vessels more homogenously compared to CD31. SNR values for the rat sample were 1.77 for the red channel (tomato lectin staining) and 1.52 for the green channel (CD31 staining). For the pig samples, the SNR values were 1.35 and 1.38 for the tomato lectin and CD31 stains, respectively. Binary masks of the green and red channels for each image were determined by thresholding (Figure S1), and a DICE score was calculated to compare the two masks. The rat image had a DICE score of 0.575, and the pig image had a DICE score of 0.319.

### 3.2 Delipidation Experiment

#### 3.2.1 Rat Tissue

**FIGURE 4:**
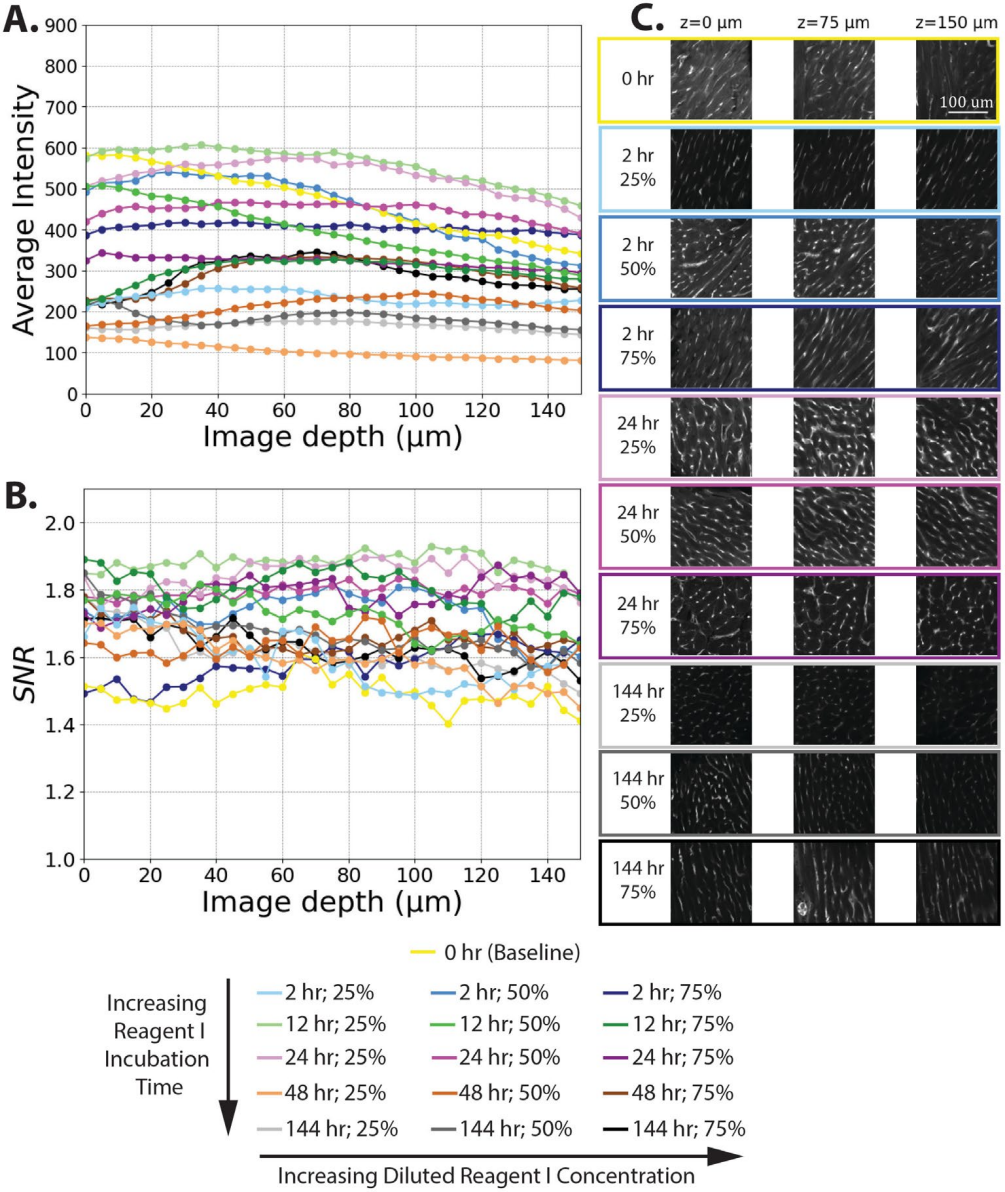
Representative results for rat myocardial tissue delipidation. (A) Average z‐slice intensity vs. image depth. (B) SNR vs. image depth. (C) representative 512 × 512 raw images for the 0‐h baseline and 12, 24 and 144 h Reagent I incubation times obtained at z‐depths of 0, 75, and 150 mm. Experimental condition denoted by color with CUBIC Reagent I time indicated by base color (2 h in blue, 12 h in green, 24 h in purple, 48 h in orange, and 144 h in gray/black) and concentration indicated by color intensity (lightest for 25% and darkest for 75%).

Myocardial tissue sections were cleared with 25%, 50%, and 75% CUBIC Reagent I concentrations during the diluted Reagent I incubation phase. The total Reagent I incubation time (including both diluted and non‐diluted concentrations) was set to 2, 12, 24, 48, or 144 h. Generally, samples showed a linear decrease in average intensity and stable SNR over the 150 μm imaging depth (Figure 4). For each sample, a percent relative intensity was calculated as the percent of average intensity at an imaging depth of 150 μm relative to the average intensity at a depth of 0 μm. The percent relative intensities for all samples were at least 55%, with a value of 58.8% for the baseline sample intensity and all other values shown in Table 2. A two‐way ANOVA on percent relative intensity showed no statistically significant effect of Reagent 1 incubation times or diluted Reagent I concentrations on percent relative intensity. For SNR, inclusion of Reagent I delipidation generally improved SNR values compared to the baseline sample without Reagent I incubation (yellow line in Figure 4b). Moderate Reagent I incubation times of 12 and 24 h (green and purple lines, respectively) were highest across the entire imaging depth. Table 3 displays SNR values for samples treated with Reagent I at imaging depths of 0 and 150 μm. Two‐way ANOVAs were conducted to compare the effect of Reagent I incubation time and diluted concentration on SNR values at imaging depths of 0 μm and 150 μm. For an imaging depth of 0 μm, no statistically significant difference in SNR values was found when comparing total incubation times or concentrations during the diluted Reagent I phase (*p* = 0.112 and *p* = 0.697, respectively). For the imaging depth of 150 μm, Reagent I incubation time has a statistically significant effect on SNR (*p* = 0.009), but diluted Reagent I concentration did not (*p* = 0.496). Pairwise comparisons with a Tukey HSD test revealed significant differences in SNR for incubation times of 12 versus 144 h (*p* = 0.038), 24 versus 48 h (*p* = 0.031), and 24 versus 144 h (*p* = 0.013). Results from ANOVA analysis and corresponding Tukey HSD tests for the rat analysis are shown in Tables S1 and S2 for an imaging depth of 0 μm and Tables S3 and S4 for an imaging depth of 150 μm.

**TABLE 2:** Percent relative intensities (100% × Intensity at 150 μm/Intensity at 0 μm) for rat and pig samples in delipidation experiments. All values are reported as a percentage.

Rat					Pig				
	25%	50%	75%	avg ± stdev		25%	50%	75%	avg ± stdev
2 h	108.2	63.4	100.2	90.6 ± 23.4	2 h	39.6	39.0	37.6	38.7 ± 1.0
12 h	79.8	57.0	125.2	87.3 ± 34.7	12 h	63.5	48.2	64.4	58.7 ± 9.2
24 h	85.0	93.1	90.5	89.5 ± 4.1	24 h	55.2	75.1	75.4	68.6 ± 11.6
48 h	59.4	123.8	113.2	98.8 ± 34.5	48 h	31.2	87.0	66.2	61.5 ± 28.2
144 h	89.8	72.6	118.8	93.7 ± 23.3	144 h	42.2	51.9	45.5	46.6 ± 4.9
avg ± stdev	84.4 ± 17.6	82.0 ± 27.1	1.10 ± 14.1		avg ± stdev	60.2 ± 20.0	46.4 ± 12.9	57.8 ± 15.7	

**TABLE 3:** SNR for rat samples in delipidation experiments at 0 and 150 μm.

*Z* = 0 μm					*Z* = 150 μm				
	25%	50%	75%	avg ± stdev		25%	50%	75%	avg ± stdev
2 h	1.662	1.735	1.492	1.630 ± 0.125	2 h	1.638	1.602	1.651	1.630 ± 0.025
12 h	1.847	1.767	1.890	1.835 ± 0.062	12 h	1.785	1.638	1.777	1.733 ± 0.082
24 h	1.837	1.778	1.730	1.782 ± 0.054	24 h	1.762	1.786	1.789	1.778 ± 0.015
48 h	1.699	1.642	1.777	1.706 ± 0.068	48 h	1.450	1.629	1.639	1.573 ± 0.106
144 h	1.780	1.849	1.713	1.781 ± 0.068	144 h	1.493	1.582	1.530	1.535 ± 0.045
avg ± stdev	1.765 ± 0.082	1.754 ± 0.075	1.720 ± 0.145		avg ± stdev	1.625 ± 0.152	1.647 ± 0.081	1.677 ± 0.108	

#### 3.2.2 Pig Tissue

**FIGURE 5:**
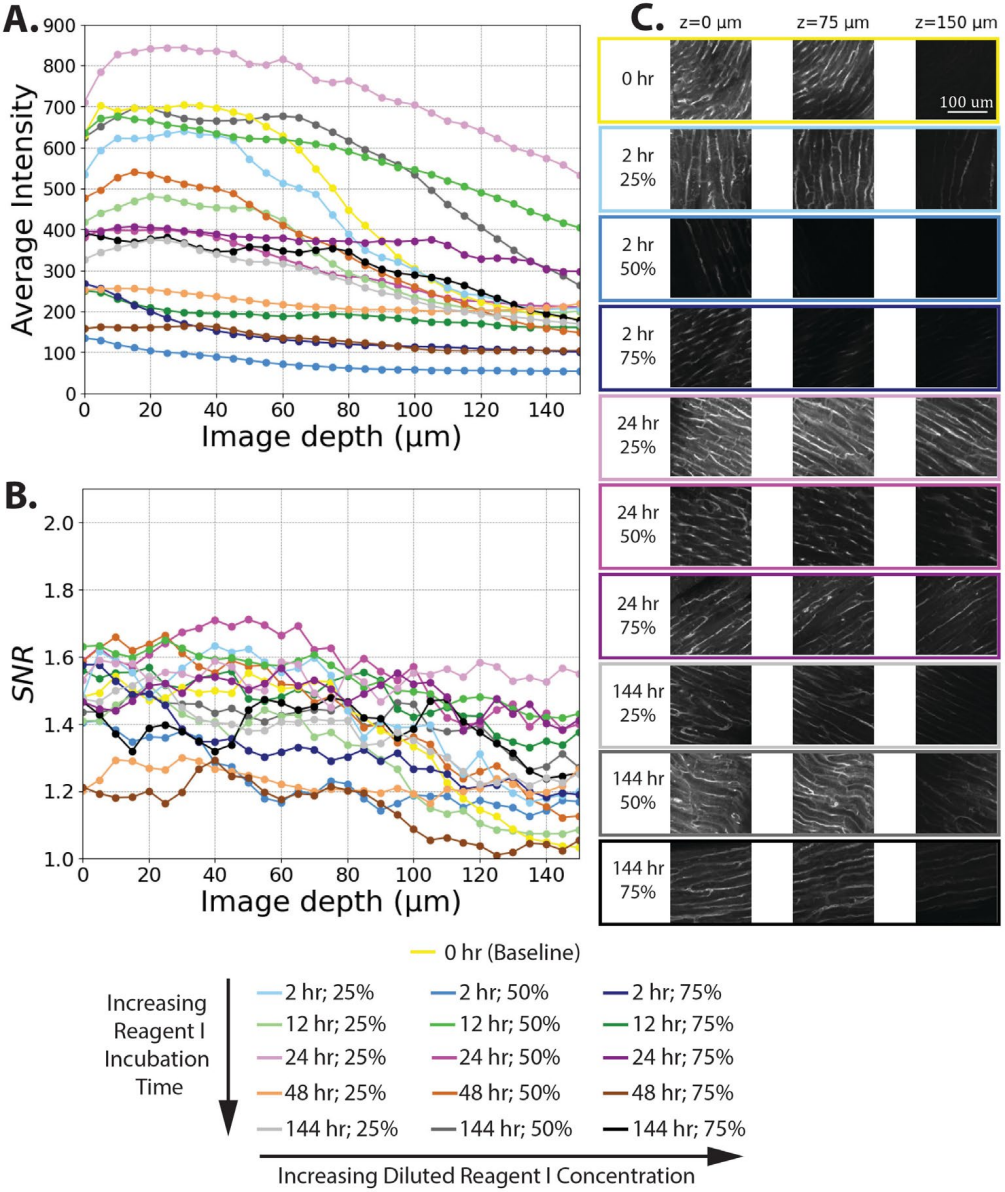
Representative results for pig myocardial tissue delipidation. (A) Average z‐slice intensity vs. image depth. (B) SNR vs. image depth. (C) representative 512 × 512 raw images for the 0‐h baseline and 12‐h, 24‐h and 144‐h Reagent I incubation times obtained at z‐depths of 0, 75, and 150 mm. Experimental condition denoted by color with CUBIC Reagent I time indicated by base color (2 h in blue, 12 h in green, 24 h in purple, 48 h in orange, and 144 h in gray/black) and concentration indicated by color intensity (lightest for 25% and darkest for 75%).

The same clearing conditions, varying diluted Reagent I concentration, and total Reagent I incubation time were tested in pig myocardial samples. The no‐Reagent I baseline sample intensity had a percent relative intensity of 28.6%, and Table 2 also displays the percent relative intensities for the Reagent I treated pig samples. A two‐way ANOVA for percent relative intensity showed no statistically significant differences comparing Reagent 1 incubation times or concentrations. Generally, the pig samples showed lower values for percent relative intensities compared to the rat samples. Trends of average intensity and SNR across imaging depth are shown in Figure 5. Notably, the baseline sample (yellow line) showed a rapid drop in average intensity between imaging depths of 60–100 μm. SNR values for the baseline sample also show a noticeable drop for imaging depths greater than 80 μm. Further, the inclusion of CUBIC delipidation steps with Reagent I incubation did not consistently improve SNR values compared to the baseline, as had been observed for the rat samples. Table 4 displays SNR values for pig samples treated with Reagent I at imaging depths of 0 and 150 μm. Moderate Reagent I incubation times of 24 h produced the highest SNR values at these deeper imaging depths. Two‐way ANOVAs did not show statistically significant differences in SNR values when comparing total incubation times or concentration during the diluted Reagent I phase at imaging depths of 0 μm (*p* = 0.312 and *p* = 0.377, respectively) and 150 μm (*p* = 0.954 and *p* = 0.075, respectively). The largest difference in SNR values was observed between samples with total Reagent I incubation times of 24 and 48 h; however, this comparison did not meet the threshold for significance in the Tukey HSD test (*p* = 0.066). Results from ANOVA analysis and corresponding Tukey HSD tests for the pig analysis are shown in Tables S5 and S6 for an imaging depth of 0 μm and Tables S7 and S8 for an imaging depth of 150 μm.

**TABLE 4:** SNR for pig samples in delipidation experiments at 0 and 150 μm.

Z = 0 μm					*Z* = 150 μm				
	25%	50%	75%	Avg ± stdev		25%	50%	75%	Avg ± stdev
2 h	1.479	1.405	1.576	1.487 ± 0.086	2 h	1.199	1.169	1.189	1.185 ± 0.015
12 h	1.409	1.630	1.560	1.533 ± 0.113	12 h	1.086	1.430	1.376	1.297 ± 0.185
24 h	1.530	1.587	1.466	1.528 ± 0.060	24 h	1.549	1.399	1.412	1.454 ± 0.083
48 h	1.202	1.584	1.212	1.33 ± 0.218	48 h	1.267	1.126	1.053	1.149 ± 0.109
144 h	1.474	1.436	1.466	1.459 ± 0.020	144 h	1.253	1.270	1.254	1.259 ± 0.010
Avg ± stdev	1.419 ± 0.129	1.528 ± 0.101	1.456 ± 0.146		Avg ± stdev	1.271 ± 0.171	1.279 ± 0.135	1.257 ± 0.145	

### 3.3 TrueBlack Experiment

**FIGURE 6:**
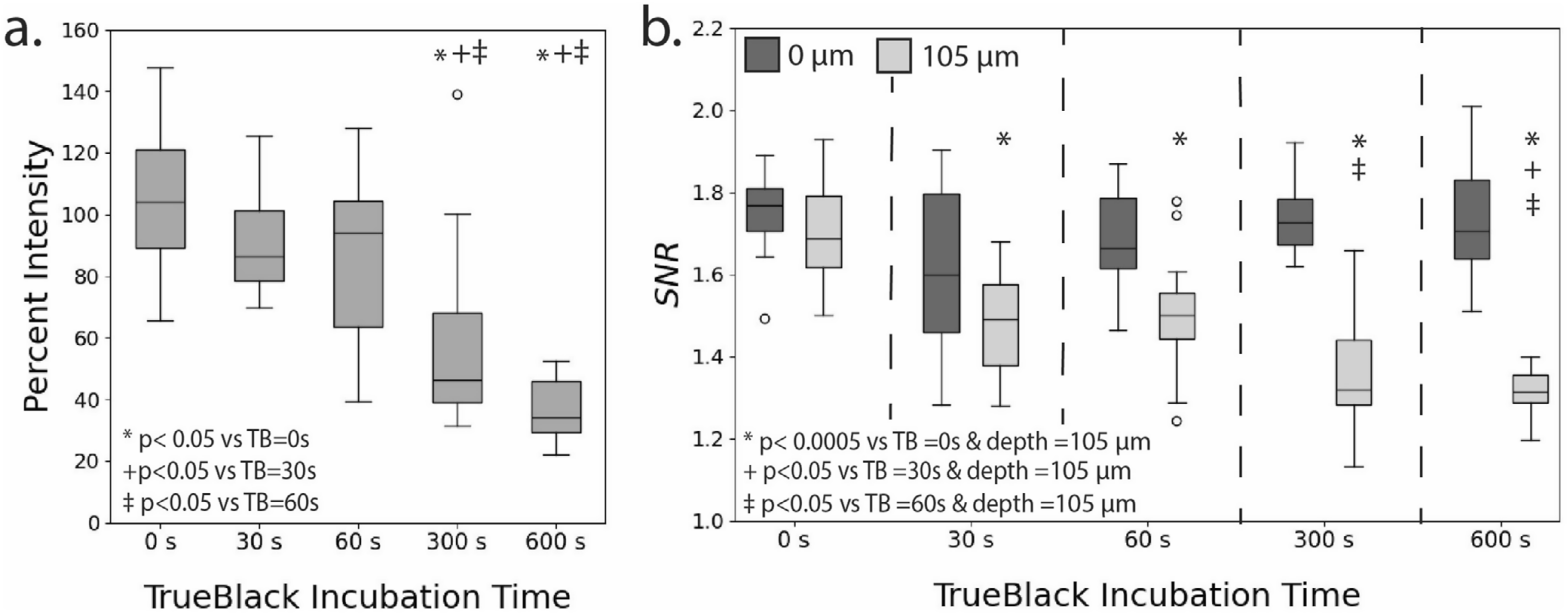
Results from the rat delipidation and TrueBlack experiments. (a) Percent relative intensities for different TrueBlack quencher incubation times (0, 30, 60, 300, and 600 s). (b) SNR values at *z* = 0 mm (dark gray) and at *z* = 105 mm (light gray) for different quencher incubation times. Trends show SNR values at *z* = 0 mm are not heavily impacted by TrueBlack incubation time, however percent relative intensities and SNR values at *z* = 105 mm decrease with increasing incubation time.

**FIGURE 7:**
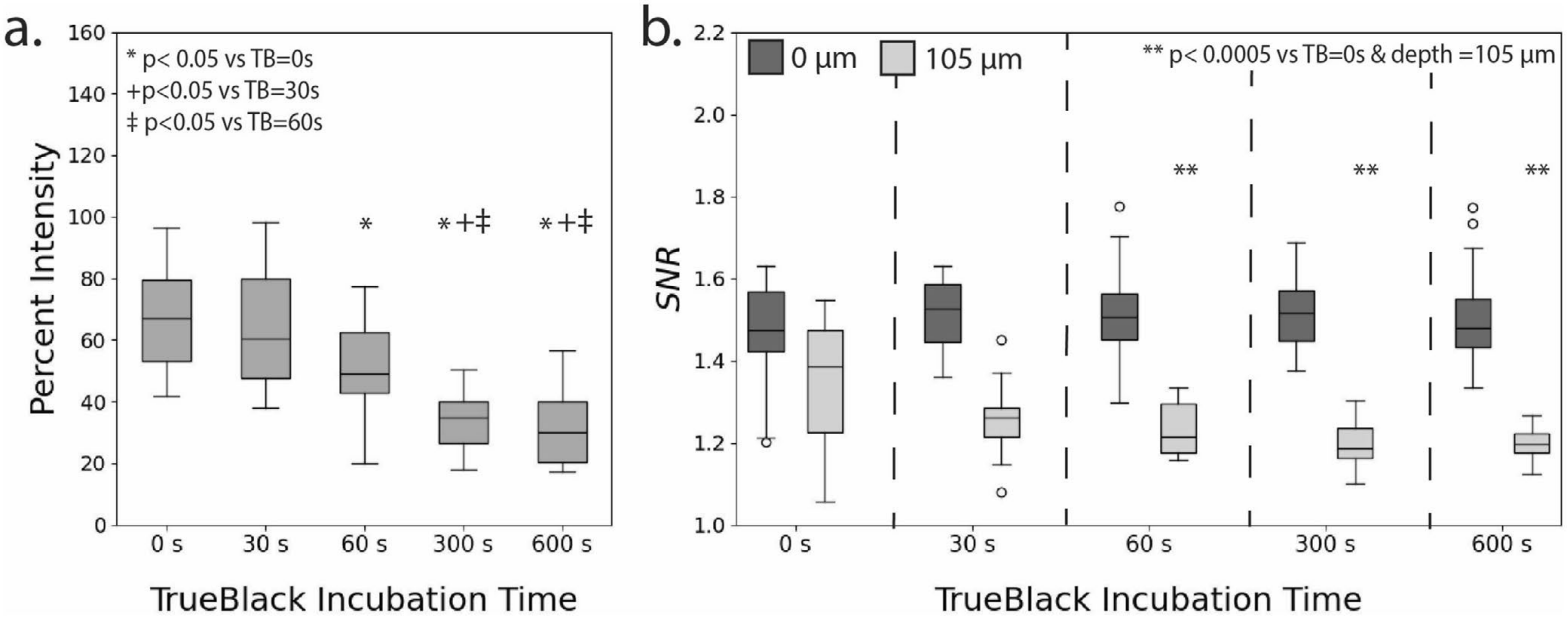
Results from the pig delipidation and TrueBlack experiments. (a) Percent relative intensities for different TrueBlack quencher incubation times (0, 30, 60, 300, and 600 s). (b) SNR values at *z* = 0 mm (dark gray) and at *z* = 105 mm (light gray) for different quencher incubation times. Trends show SNR values at *z* = 0 mm are not heavily impacted by TrueBlack incubation time, however percent relative intensities and SNR values at *z* = 105 mm decrease with increasing incubation time.

The impact of sample incubation time in autofluorescence quenching reagent TrueBlack was studied in combination with the previous experimental variables (Reagent I percent dilution and incubation time). Trends for imaging depth versus intensity and for all samples are shown in Figures S2 and S3. Due to surface roughness, not all samples had a complete imaging depth of 150 μm, and the shallowest sample had a maximal imaging depth of 105 μm. Therefore, comparisons were performed between imaging depths of 0 and 105 μm for both rats and pigs. Statistical comparisons only included samples that received a Reagent I incubation and did not include the baseline no‐Reagent 1 samples for each TrueBlack incubation. Boxplots showing the median and distribution of percent relative intensity and SNR values for each imaging depth are presented for rats (Figure 6) and pigs (Figure 7).

One‐way ANOVAs and Tukey HSD tests were performed for both rats and pigs to identify the effect of TrueBlack incubation time on percent relative intensity, which was defined based on the ratio of average z‐slice intensities at an imaging depth of 105 and 0 μm. For both rat and pig samples, TrueBlack incubation time did have a statistically significant main effect on percent relative intensity. Furthermore, pairwise comparisons from the Tukey HSD test indicated that percent relative intensities for samples treated with TrueBlack incubations of 300 and 600 s significantly differed from samples with 0, 30, and 60 s incubations.

A similar set of analyses was performed to identify the effect of TrueBlack incubation time on SNR values at imaging depths of 0 and 105 μm for rats and pigs. At imaging depths of 0 μm, TrueBlack incubation time did not have a statistically significant main effect on SNR values for rat and pig samples. However, the ANOVA for imaging depth of 105 μm in both rat and pig samples showed significant differences in SNR between TrueBlack incubation time groups (*p* = 2.7 E‐12 and *p* = 6.60 E‐5, respectively). For the rat samples, a Tukey HSD test revealed significant differences in SNR values at an imaging depth of 105 μm between all levels of TrueBlack incubation compared to the control samples (0 s of incubation). Additional comparisons between incubation times of 30 versus 300, 60 versus 300, and 60 versus 600 s were significant for the rat tissue samples at 105 μm. For the pig tissue samples, significant differences were observed for the control samples compared to samples with incubation times of 60, 300, and 600 s. Results from ANOVA and the Tukey HSD test comparing percent relative intensity and SNR between different TrueBlack incubation times are included in Tables S9 and S10 for rats and Tables S11 and S12 for pigs.

### 3.4 Comparative Autofluorescence Quenching Experiment

**FIGURE 8:**
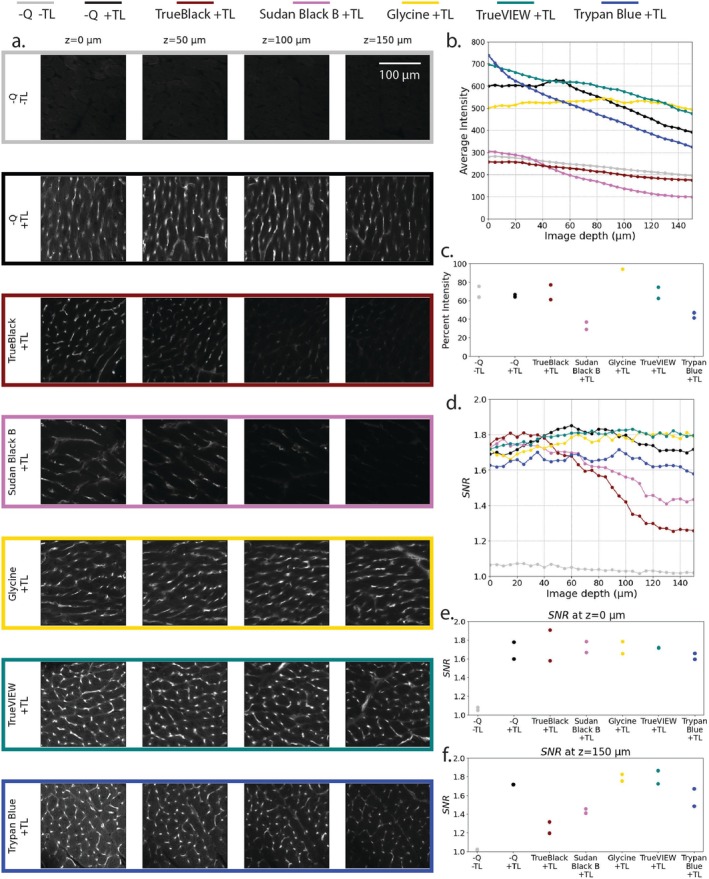
Rat myocardial tissue sections treated with various autofluorescence quenching reagents. (a) z‐plane slices at depths of 0, 50, 100, and 150 μm. The first and second row corresponds to a control sample with no quenchers and without and with no tomato lectin (−Q/−TL and −Q/+TL). Profiles of average intensity and SNR versus imaging depth (b, d) are averages of two samples for each condition. Scatter plots of percent relative intensity (c) and SNR values at 0 and 150 μm (e, f) show the two samples for each condition.

**FIGURE 9:**
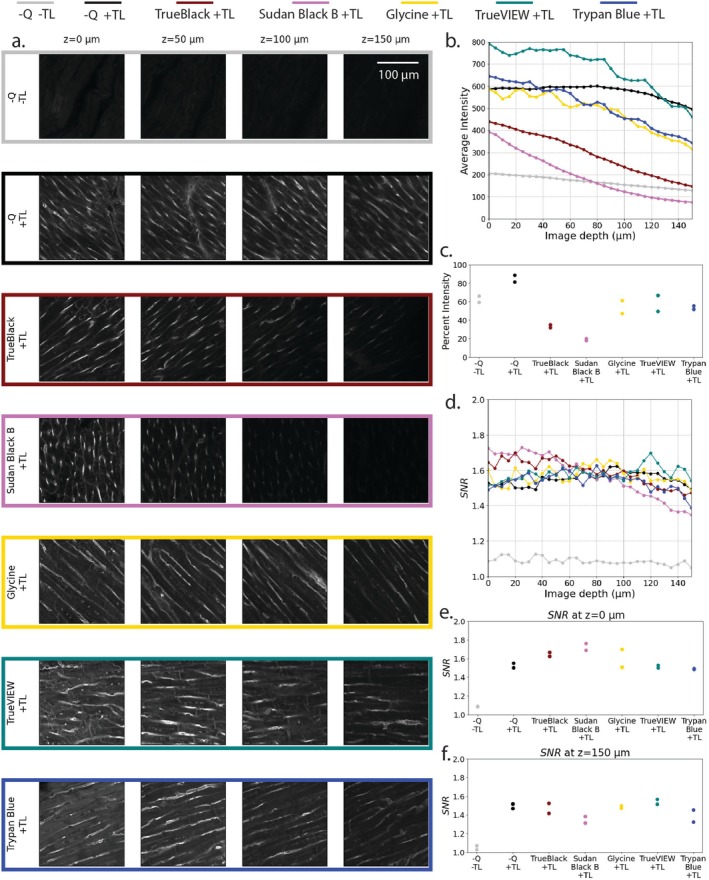
Pig myocardial tissue sections treated with various autofluorescence quenching reagents. (a) z‐plane slices at depths of 0, 50, 100, and 150 μm. The first and second row corresponds to a control sample with no quenchers and without and with no tomato lectin (−Q/−TL and −Q/+TL). Profiles of average intensity and SNR versus imaging depth (b, d) are averages of two samples for each condition. Scatter plots of percent relative intensity (c) and SNR values at 0 and 150 μm (e, f) show the two samples for each condition.

A comparison between different autofluorescence quenchers (Sudan Black B, TrueBlack, Glycine, TrueVIEW, and Trypan Blue) was performed by keeping the CUBIC Reagent I conditions consistent between samples. For both rat and pig myocardial samples, a diluted Reagent I concentration of 50% was used. Samples were allowed to incubate in diluted and 100% Reagent I for 12 h each, for a total Reagent I incubation time of 24 h. Results for rat and pig tissues are shown in Figures 8 and 9, respectively.

Two types of controls were used. The first type of control samples with no quenchers and no tomato lectin (−Q/−TL, gray lines) offers a measure of the tissue autofluorescence. The second type of control samples with no quenchers but with tomato lectin (−Q/+TL, black lines) shows increased intensity SNR compared to the −Q/−TL samples and offers a baseline for assessing the performance of the quenchers.

Figures 8 and 9, shows representative images for both controls and each of the five quenchers (panel a). Trends of average intensity (panel b) and SNR (panel d) versus imaging depth represent the average of the two independent samples for each condition. Panels c, e, and f display value for the two independent samples of each condition for percent relative intensity (panel c), SNR at imaging depth of 0 μm (panel e), and SNR at imaging depth of 150 μm (panel f). For some samples such as the Glycine +TL samples in 8c or the Trypan Blue +TL samples in 9e, measurements for the duplicates of the experimental condition were similar and the corresponding points overlapped in the respective figures.

Statistical analyses (one‐way ANOVAs) were performed to examine the effect of quencher type on the values of percent relative intensity and SNR at imaging depths of 0 and 150 μm. Tukey HSD tests were performed for pairwise comparisons. Statistical analysis focused on the presence and type of quencher and did not include the −Q/−TL baseline samples.

#### 3.4.1 Rat Tissue

Compared to the −Q/+TL baseline, glycine samples had higher percent relative intensities and Sudan Black B had lower percent relative intensities. Pairwise comparisons for both quenchers compared to the −Q/+TL baselines were identified as statistically significant using the Tukey HSD test. Additionally, compared to the −Q/+TL baseline, none of the autofluorescence quenchers significantly improved SNR at imaging depths of 0 or 150 μm. TrueVIEW (green lines) and Glycine (yellow lines) maintained high values of SNR throughout the total imaging depth. TrueBlack (dark red lines) and Sudan Black B (pink lines) show lower intensity and reduced SNR at large depths. An ANOVA revealed no statistically significant difference appeared between any of the types of quenchers for imaging depths of 0 μm. However, at an imaging depth of 150 μm, significant differences were observed (*p* = 0.00251). TrueBlack samples showed statistically significant differences compared to the −Q/+ TL baseline (*p* = 0.00867), Trypan Blue (*p* = 0.0463), TrueVIEW (*p* = 0.00385), and Glycine (*p* = 0.00407) samples. Further, Sudan Black B samples showed significant differences compared to samples treated with TrueVIEW (*p* = 0.0275) and Glycine (*p* = 0.0295). Results from ANOVA and Tukey HSD test comparing percent relative intensity and SNR values between the −Q/+ TL baseline and 5 quencher types are included in Tables S13 and S14 for the rat samples.

#### 3.4.2 Pig Tissue

For pig tissue samples, the −Q/+TL− baseline samples had the highest values for percent relative intensity. Pairwise comparisons revealed significant differences for TrueBlack, Sudan Black B, Trypan Blue, and Glycine samples compared to the −Q/+TL− baseline samples. Additionally, for the pig tissue samples, a general trend of lower SNR values compared to the rat tissue samples was observed. One‐way ANOVAs performed on the pig samples revealed no statistically significant differences between the quencher types on SNR values at imaging depths of 0 and 150 μm (*p* = 0.0545 and *p* = 0.0921, respectively). At an imaging depth of 0 μm, Sudan Black B samples had a non‐statistically significant trend of higher SNR values relative to the −Q/+TL baseline (*p* = 0.121), Trypan Blue (*p* = 0.0621), and TrueVIEW (*p* = 0.0972). At imaging depths of 150 μm, Sudan Black B samples had lower, but not statistically significant SNR values compared to the −Q/+TL baseline and other quencher types. Results from ANOVA and Tukey HSD test comparing percent relative intensity and SNR values between the −Q/+TL baseline and 5 quencher types are included in Tables S15 and S16 for pig samples.

### 3.5 Segmentation, Skeletonization, and Morphometrical Quantification

**FIGURE 10:**
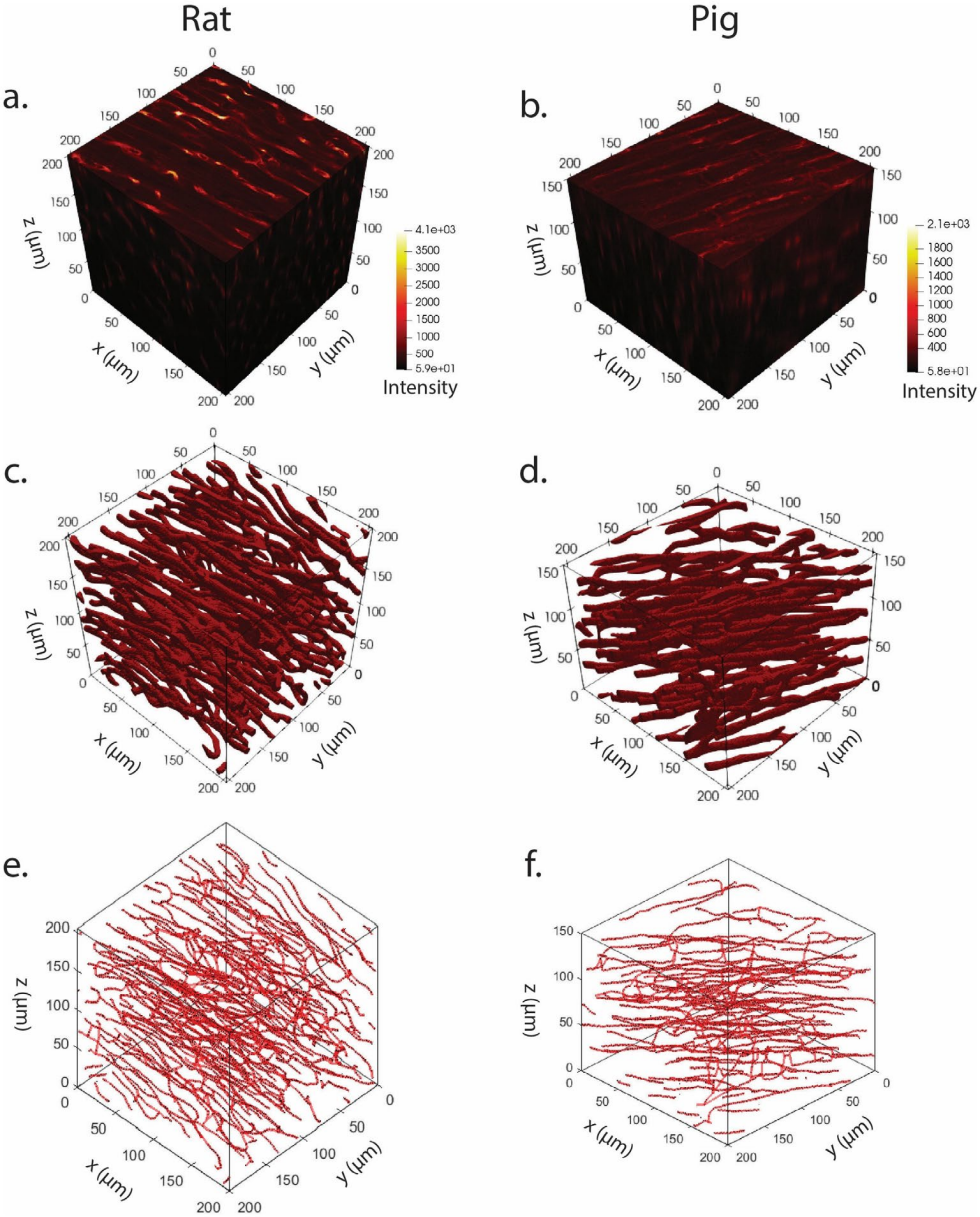
High resolution volumetric images (a and b for rat and pig tissue, respectively), corresponding segmented images (c, d for rat and pig tissue, respectively), and skeletonizations (e, f for rat and pig, respectively).

Higher resolution images were taken of rat and pig myocardial samples (processed with 24 h of total Reagent I incubation time, 25% diluted Reagent I concentration, and no autofluorescence quenchers) and with an x‐y resolution of 0.4 μm/pixel and z‐slices spaced 0.975 μm apart to allow for a 3D reconstruction of the microvascular network in each image. Imaging depths of 201.875 and 150 μm were achieved for the rat and pig samples, respectively. Segmented images and skeletonizations are shown in Figure 10. Following skeletonization, graph networks were created with each capillary assigned an average diameter and total length. Distributions of vessel diameter and lengths for the high‐resolution volumetric images in Figure 10 are shown in Figure S4, and mean values are reported in Table 5 and compared to similar metrics reported in literature [14, 15, 29, 30, 49, 50].

**TABLE 5:** Comparison of morphometrical quantification from high resolution 3D images to literature.

Sample/Source	Species type	Labeling method	Capillary type	Vessel length	Vessel diameter
High resolution rat Myocardium (Figure 10a)	Rat	Immersion with tomato lectin and CUBIC	All	37.6 ± 31.3 μm	7.1 ± 2.0 μm
High resolution pig Myocardium (Figure 10b)	Pig	Immersion with tomato lectin and CUBIC	All	56.4 ± 49.8 μm	7.1 ± 2.3 μm
Vigneshwaran et al. [29]	Rat	Perfusion with tomato lectin and CUBIC	All	Range from 6 to 300 μm	6.5 ± 1.2 μm
Olianti et al. [30]	Rat	Vascular perfusion with BSA‐FITC gel	All	NA	~0.8–1.5 μm
Kassab et al. [15]	Pig (LV)	Silicone elastomer casts	C0 vessels (C_00_)	54.5 ± 43.0 μm	5.7 ± 1.2 μm
			Cc vessels (C_cc_)	21.1 ± 15.5 μm	5.5 ± 1.4 μm
Kaneko et al. [49]	Human (LV)	Serial sectioning stained with modified Masson technique	All	61.5 ± 41.6 μm	NA
Anderson and Anderson [50]	Dog	Methyl methacrylate casts	All	NA	5.5 μm
Bassingthwaighte et al. [14]	Dog	Silicone elastomer casts	All	NA	Uncorrected: 3.1 μm Corrected: 5.5 ± 1.3 μm

## 4 Discussion

Tissue clearing and microvascular imaging techniques have progressed significantly in the last few decades, particularly in cerebrovascular imaging applications. However, for highly auto‐fluorescent tissues such as the myocardium, more research is required to understand the performance of different quenching and clearing agents. The work presented here outlines a protocol for immersion‐based vascular labeling and tissue clearing for myocardial samples using tomato lectin and Sca*l*eCUBIC reagents, respectively. The effectiveness of this protocol has been demonstrated for rat and pig tissues and for imaging depths of up to 150 μm. Furthermore, we explored if the addition of autofluorescent quenchers improves SNR and increases the imaging depth.

### 4.1 Tomato Lectin and CD31 Colocalization

Anti‐CD31 antibodies, also known as Platelet Endothelial Cell Adhesion Molecule‐1 (PECAM‐1), and lectins have been frequently used to label vascular endothelial cells [18, 51, 52]. However, the effectiveness of vascular labels, particularly lectins, can differ between species or with different labeling methods (e.g., perfusion versus immersion).

Our immunofluorescence labeling results demonstrated modest colocalization of *L. esculentum* tomato lectin (LEL) and CD31 signals. The DICE scores of rat and pig images were 0.467 and 0.207, respectively. For the rat sample, the red channel with tomato lectin produced a higher SNR (1.77) than the green channel with CD31 fluorescence (1.52). For the pig, both channels produced low SNR values, with the green‐fluorescent CD31 producing a higher SNR (1.38) than the red tomato lectin (1.35). Additionally, tomato lectin appeared to provide a more consistent stain for vessels while the CD31 stain produced a spottier pattern, likely due to the different molecular weights of the dyes, which affect their permeability. The unconjugated lectin weighs 71 kDa [53] and unconjugated Dylight 594 weighs ~1 kDa [54] (for a total weight of ~72 kDa), while the primary and secondary CD31 antibodies utilized in this study weigh ~120 kDa [55, 56] and ~150 kDa [57], respectively. For these reasons, tomato lectin was the only labeling agent tested in subsequent experiments.

A previous study by Zhu et al. using tail‐vein injection found that tomato‐lectin provided higher signal‐to‐background ratios than CD31 for mouse cerebral tissue [17]. Moreover, comparing three types of lectins, Battistella et al. found that LEL produced the highest SNR in immersion‐based histologically stained mice brains and SNR increased with decreasing concentration of LEL. Furthermore, for cardiac muscle, their results showed that immersion‐based histological staining with low concentrations of LEL (5 μg/mL) produced similar SNR compared to 50 μg/mL Wheat Germ Agglutinin (WGA) perfusion [51]. In this study, we used a LEL tomato‐lectin concentration of 10 μg/mL. Although higher SNRs may be attainable at the surface with reduced tomato‐lectin concentration, the impact of labeling concentration on imaging depth is unknown and worth investigating. This was outside the scope of this study.

### 4.2 Delipidation Experiment

The Sca*l*eCUBIC protocol uses two clearing reagents. CUBIC Reagent I targets the removal of lipids and pigments from the tissue (delipidation and decolorization), whereas CUBIC Reagent II increases the transmittance of the sample with RI matching, see Susaki et al. [25]. In this work, the impact of different conditions (incubation time and concentration) of the delipidation step on image quality was studied. For RI matching, we elected to keep Reagent II incubation times constant throughout this experiment. Adjustments to incubation time in Reagent II were assumed to be insignificant to image quality, given that one of the original protocols claimed samples could be stored in Reagent II for up to 2 weeks following the initial Reagent II immersion [26] and previous work had success making whole hearts sufficiently optically transparent using only treatment of Reagent I and a pre‐imaging transfer to SCALE [58]. For the delipidation steps, a pre‐treatment with 50% diluted CUBIC Reagent I improved clearing compared to direct immersion in 100% CUBIC Reagent I in Susaki et al. [25]. While designed for whole‐brain imaging on mice, the group subsequently showed these reagents could be used to clear various tissue types [26]. Methods of clearing myocardial tissue with CUBIC reagents typically involve administering CUBIC Reagent I using intracardial perfusion, multiday immersion, or both [28, 58, 59].

We studied the impact of dilution concentrations and total incubation time of CUBIC Reagent I (the combined time for diluted and full‐concentration reagents) on image quality and imaging depth in an immersion‐only protocol. For the rat tissue, loss of average z‐slice intensity across the imaging depths of 150 μm was minimal. Values of percent relative intensity for all samples were greater than 55%, and many of the cleared tissue samples had values > 85%. Some samples had percent relative intensities greater than 100%, potentially due to larger vessels at deeper imaging depths or the diffusion of clearing reagents from the other side of the sample. The surface roughness, which we observed in this study, could further the likelihood of the later explanation. Although we did not see significant differences in percent relative intensities in the rat delipidation experiment, SNR results suggested that a total incubation time of 24 h was optimal for rat tissue. Longer Reagent I incubation times of 48 and 144 h resulted in reduced SNR compared to samples with moderate Reagent I incubation times of 12 and 24 h (Figure 4, Table 2). We did not observe significant differences in SNR when comparing different concentrations of diluted Reagent I. For the pig tissue, larger sample variability, less apparent SNR trends, and lower percent relative intensities were observed. Nevertheless, samples with moderate incubation times of 24 h did have higher SNR values at deep imaging depths compared to the other samples (Figure 5). Furthermore, although not statistically significant, the 24‐h total Reagent I incubation time did have the highest average percent relative intensity in the pig delipidation experiment.

A significant finding of this study is that long incubation times in CUBIC Reagent I could harm image quality. Our results suggest that there is an optimal, moderate incubation time for CUBIC Reagent I for immersion‐based approaches and that optimizing SNR should be considered when planning the length of incubation time in protocols using CUBIC clearing reagents. Furthermore, both rat and pig tissues had the same optimal Reagent I incubation time, with the highest SNR at imaging depths of 150 μm measured in samples incubated for a total of 24 h. This suggests that the optimal incubation time may be consistent for myocardial tissue across different species if maintaining the same thickness of the tissue samples.

Many current protocols (involving perfusion and immersion) incorporate multi‐day incubation times [21, 28, 58]. Longer incubation times are likely necessary when working with thicker tissue sections, up to whole hearts in small animals. Furthermore, most protocols focus on maximizing tissue transparency rather than on producing the highest SNR for a given label. Zhu et al. [17] noted reductions in signal‐to‐background ratios of various vascular labels including intravenously applied tomato‐lectin, CD31, and a FITC‐dextran mixture comparing samples before and after clearing when using CUBIC reagents, but not when using solvent‐based clearing methods. Each clearing method has advantages and disadvantages, and different combinations of label and clearing reagents will have different interactions [24]. Comparing tissue clearing methods was outside the scope of this study. However, future research should consider other methods such as CLARITY or FASTClear, which have both been previously used for visualization of microvascular structures in myocardial tissues [2, 60].

Additionally, the order of the clearing and labeling steps may be important to consider. While perfusion‐based methods typically label vasculature prior to clearing, we chose to incorporate vascular labeling after delipidation. Delipidation disrupts cellular lipid membranes, increases tissue permeability, and can increase antibody penetration depth [61]. Sands et al. demonstrated the permeability of connexin‐43 antibodies and WGA in 0.75 mm thick sections of human right ventricular myocardial tissue previously cleared with CUBIC reagents [21]. In theory, this rationale of increased permeability may extend to the use of labeling with lectin and other non‐antibody labels. Additionally, previous results suggest that immersion in CUBIC reagents following vascular labeling reduces signal‐to‐background ratios [17]. For these reasons, tomato‐lectin incubation was performed following the delipidation step of CUBIC reagent I. It is important to note that this method is based on a tomato‐lectin labeling, and not antibody labeling. For future work with antibody labeling, which typically has higher molecular weights than lectins, permeability of the label in the tissue and optimal tissue clearing time may differ from this study.

### 4.3 TrueBlack and Comparative Autofluorescence Quenching Experiment

Tissue autofluorescence from endogenous sources such as heme, lipofuscin, flavoproteins, elastin, collagen, and NADH can increase background signal and harm SNR [32]. Additionally, formaldehyde crosslinking from PFA fixation can further contribute to the fluorescence of fixed tissue [31]. Amino‐alcohols in CUBIC Reagent I have been previously shown to aid in the elution of heme from erythrocytes and the decolorization of blood [28]. Other sources of autofluorescence can be targeted with various quenchers. Lipofuscin can be targeted with Sudan Black B or TrueBlack [39]. Two other quenching agents, TrueVIEW and Trypan Blue, have been reported to non‐specifically bind to different fluorescent components. TrueVIEW uses electrostatic interactions to target non‐lipofuscin sources of intrinsic autofluorescence, such as erythrocytes, collagen, and elastin in formalin‐fixed tissues [37]. Trypan Blue has been reported as an effective quencher for flavin mononucleotides, which fluoresce primarily in the green wavelengths [40]. Glycine is known to react with formaldehyde residues and may reduce the autofluorescence of PFA‐fixed tissues [38].

First, we examined the TrueBlack quenching agent and its effect on image quality and depth by varying TrueBlack incubation time (0, 30, 60, 300, and 600 s). A more in‐depth analysis of TrueBlack was performed since it had been reported as the best performing quencher for fixed mouse adrenal cortex tissue [36]. Additionally, previous studies using formaldehyde‐fixed myocardial tissue suggested that TrueBlack could reduce autofluorescence at the surface of the sample [32], which may improve image quality and SNR values. In this study, we did not observe any significant effect of TrueBlack incubation on SNR at imaging depths of 0 μm for either rat or pig samples. Furthermore, we observed decreases in both SNR at deeper imaging depths (105 μm) and percent relative intensity with increasing time of TrueBlack incubation. These results suggested that the inclusion of TrueBlack did not improve image quality at shallow depths and harmed image quality at deeper imaging depths.

To understand whether other quenchers may be able to outperform TrueBlack at deeper imaging depths, a comparative study across quenchers was conducted using five quenching agents: TrueBlack, Sudan Black B, Trypan Blue, Glycine, and TrueVIEW. The incubation times chosen for these quenchers were based on incubation times in other published literature [32, 36]. Relative to the −Q/+TL baseline, none of the autofluorescence quenchers significantly improve SNR for imaging depths of 0 or 150 μm (Figures 8 and 9). However, different performances were observed among the quenchers with respect to imaging depth.

TrueBlack and Sudan Black B show general trends of lower intensity throughout the imaging depth and reduced SNR at deep imaging depths compared to −Q/+TL baseline samples. When only considering decreases in autofluorescent signal, Zhang et al. show that Sudan Black B and TrueBlack perform best in suppressing autofluorescence in mouse myocardial tissue [32]. For both of these quenchers, reductions in average signal intensity across the entire imaging depth suggest a decrease in autofluorescent signal. However, for the rat samples, neither Sudan Black B nor TrueBlack significantly increased SNR values at shallow imaging depths. For the pig tissue samples, Sudan Black B did show a non‐statistically significant trend of improved SNR at imaging depths of 0 μm. The comparative quencher experiments showed trends of reduced percent relative intensity and reduced SNR for samples treated with Sudan Black B compared to no quencher (−Q/+TL) controls for rats and pigs. Additionally, compared to −Q/+TL controls, the TrueBlack treated rat tissue samples showed reduced SNR values at imaging depth of 150 μm and TrueBlack treated pig tissue samples showed reduced percent relative intensity. One explanation for these trends of reduced percent relative signal intensity and reduced SNR at deeper imaging depths is that both TrueBlack and Sudan Black B are dyes [62]. Following the quencher incubation step and subsequent wash steps, samples treated with TrueBlack and Sudan Black B maintain a dark blue or black coloring (Figures S5 and S6). This coloration increases light absorption and decreases light penetration into the tissue.

Trypan Blue is also a dye [62]; however, the treated samples appeared a light and semi‐transparent blue compared to the TrueBlack and Sudan Black B treated samples. This light coloring could explain the trend of slight reductions in SNR values at deep imaging depths and percent relative intensities for Trypan Blue samples compared to the −Q/+TL baselines. Trypan Blue has been noted to suppress autofluorescence and potentially improve SNR for images acquired with green wavelength [40]. For images acquired at other wavelengths, the improvements in SNR may not be as notable. The inclusion of Trypan Blue quenching allowed for imaging depths up to 100 μm and is worth considering in future myocardial studies where green fluorophores are used.

TrueVIEW and Glycine are not dyes and could therefore potentially improve SNR at deeper imaging depths, compared to other dye‐based quenching agents. TrueVIEW initially rendered the tissues slightly blue, and a reduction in color intensity was noted after incubation in Reagent II (Figure S6). In the rat tissue experiment, samples treated with glycine did have a significantly higher percent relative intensity compared to the −Q/+TL baseline samples. However, for both rat and pig samples, no significant differences in SNR could be observed between Glycine or TrueVIEW and the −Q/+TL baseline samples at either the 0 or 150 μm imaging depth. Other papers have reported moderate reductions in autofluorescence with Tris‐Glycine in formalin‐fixed human respiratory tissue [62]. Tris has also been reported as a formaldehyde quenching reagent and could be considered in future quenching studies [38].

### 4.4 Image Analysis Methods

Quantification of SNR requires identifying regions of interest (which typically present a high signal) compared to background regions (typically displaying a low signal). This identification could have been done through automated vessel segmentation algorithms [29, 63], or through manual identification of regions of interest by a skilled operator [17, 64]. Developing a segmentation algorithm was outside the scope of this work. Furthermore, we wanted to limit potential biases introduced by manual workflows. Therefore, in this work, we developed an automated workflow for SNR quantification that relies on the definition of bright and dark regions within each z‐slice. Due to the “vignetting” effect seen in digital microscopes where intensity decreases near the outer edges of an image, each z‐slice was cropped from a 1024 × 1024 to a 512 × 512 pixel region at the center of the image. Search boxes were used to automatically identify 15 pairs of bright (vessel) and dark background (tissue) regions within each z‐slice. The size and shape of search boxes remained consistent for all images. Bright vascular regions were identified using a 5 × 5 pixel (2 × 2 μm) search box, chosen based on the assumption that the minimal capillary diameter is 3 μm [65]. This ensures that bright vascular regions identified by the search box could fit entirely within a capillary and minimizes any potential diameter‐dependent biases in the image analysis. A larger 20 × 20 pixel (8 × 8 μm) search box was chosen for the dark tissue region to sample a more expansive area representative of tissue heterogeneity while remaining well below the average myocyte diameter of ~20 μm [66, 67]. Bright regions were identified successively, with each new bright region placed at least 75 pixels away from all previously identified bright regions. This strategy ensured sampling from multiple vessels and tissue locations across each z‐slice. Alterations to any of these parameters (number and size of sampling regions) may alter the overall SNR values.

### 4.5 Experimental Design

This study had two primary goals: (1) to assess how delipidation and quenching (TrueBlack) stages of the protocol altered tissue clearing and image quality through factorial design of key experimental parameters, and (2) to compare different quenchers within myocardial tissues. To achieve each goal, numerous myocardial samples were cleared in parallel with staggered start times to ensure concurrent protocol completion, followed by imaging within a maximal 48‐h time window. This approach eliminated temporal batch effects that occur with confound microscopy and optical clearing studies [68]. These time constraints limit the ability to perform larger numbers of replicates, and statistical assumptions of normality on homogeneity of variances on the samples could not be tested.

Furthermore, due to limited replication, we sought to minimize the sample size (*n* = 3 for rats and *n* = 1 for pig) and biological variability within this study. Three rat hearts were required to provide enough left ventricular free wall tissue for the different experiments. The delipidation and TrueBlack experiments were performed with myocardial tissue from two rats euthanized on the same day. Myocardial tissue from the third rat was used for the Comparative Autofluorescence Quenching experiments. Overall, our experimental design prioritized minimizing experimental variability over replication, reflecting a deliberate methodological trade‐off necessitated by resource constraints and tissue processing requirements.

### 4.6 Impact of Species and Disease State on Tissue Clearing

The immersion method for clearing myocardial tissue and labeling vasculature was tested for both small (rats) and large (pigs) animals. Rat myocardial tissue was selected to compare with perfusion‐based clearing from prior literature [10, 21]. Conversely, pig myocardial tissue was used due to its similarity to human myocardial tissue.

To limit excess animal experimentation and to initially test our protocol, we opted to use spare myocardial tissue from other studies. The rat myocardial tissues were presumed to be healthy. However, pig myocardial samples may not reflect a healthy phenotype. The Ossabaw pig tissue samples were acquired from an animal that underwent a ventricular‐pacing protocol for 2 weeks, designed to induce heart failure with a preserved ejection fraction. Previous histology results from a 4‐week pacing protocol revealed increased interstitial fibrosis and decreased capillary density [35].

In this study, we observed that pig tissue samples had higher average intensities, lower SNRs, and lower percent relative intensity compared to the rat tissue samples. This was observed across all experiments, including the tomato lectin and CD31 colocalization experiment, which did not include CUBIC clearing reagents. We are unable to determine whether differences in average intensity or SNR are the result of species differences in response to vascular labels, tissue autofluorescence, or due to the pacing protocol for the pig. Potential decreases in microvascular density or increases in interstitial fibrosis in the disease phenotype may reduce SNR compared to healthy pig myocardial tissue. Immersion‐based labeling and clearing techniques use microvasculature as conduits to aid in the diffusion of tissue labels or clearing reagents. Decreases in microvascular density or microvascular remodeling may hinder the transport of these materials through the tissue. Moreover, baseline autofluorescence or the effectiveness of tissue clearing reagents may differ between fibrotic and healthy myocardial tissue. Previous work comparing the clearing time of spontaneous hypertensive rats and controls reported that sections of control hearts were optically clear in 5–7 days, while hypertensive samples took 10–14 days [10].

## 5 Limitations and Future Work

Intra‐animal replicates (different sections of tissue from the same animal with the same experimental condition) were only performed for the *Comparative Autofluorescence Quenching Experiment* (*n* = 2). Although we measured similar intensities and SNR for these intra‐animal replicates, we did not fully assess the effect of regional microvascular differences or myocardial fiber direction on image quality. Additional intra‐animal replicates or imaging of multiple regions from each tissue slice would minimize the impact that large vessels or regions with lower microvascular density may have on the analysis.

Additionally, biological replication (tissues from different animals within a species and the same experimental condition) was not analyzed within this study. Anatomical variations in microcirculatory structure between animals or tissue regions should be considered for future studies focused on morphological analysis.

Future studies should consider how regional microvascular differences and fiber orientation affect tissue clearing and microvascular imaging using intra‐animal replicates. Furthermore, additional work is required to compare the effectiveness of this protocol for healthy and diseased myocardial tissue of the same species. This future work will use biological replicates and explore the physiological variation of the microvascular structure in health and disease for both porcine models and donor tissues.

## 6 Conclusion

In conclusion, we presented an immersion‐based tissue clearing protocol and demonstrated that it could successfully clear myocardial tissue and image microvascular structures up to 150 μm deep. The protocol was demonstrated for both pig and rat myocardial tissues. Image quality was assessed using both average z‐slice intensities and SNR, with an automatic analysis of SNR providing an unbiased analysis. Our findings suggest that moderate CUBIC Reagent I incubation times of 24 h may provide optimal image quality. When comparing various autofluorescence quenching reagents, results showed that TrueBlack and Sudan Black B harm imaging depth, and that TrueVIEW, Glycine, and Trypan Blue did not significantly harm SNR or percent relative intensities at imaging depths of 150 μm. The ability of these quenchers to target different sources of autofluorescence warrants further investigation. Overall, we believe this work provides a rigorous foundation for the optimization of immersion‐based protocols for myocardial tissue clearing. Using the results of this work, future studies will focus on the quantification of anatomical and topological coronary microvascular differences due to sex and disease states for different species.

## 7 Perspectives

We present and optimize an immersion‐based protocol for vascular labeling and tissue clearing using tomato lectin labeling and CUBIC clearing reagents, respectively. Success of the protocol was demonstrated with imaging depths up to 150 μm in rat and pig myocardial tissue. This protocol enables microvascular analysis for myocardial tissue from large animal studies or donor samples without the need for perfusion‐based approaches.

## Disclosure

Permission to Reproduce Material From Other Sources: Figure 1 was created with BioRender.com and is included here with permission under BioRender's Academic License.

## Ethics Statement

All the protocols involving animals conformed to the National Institutes of Health Guide for the Care and Use of Laboratory Animals and were approved by the University of Michigan Animal Research Committee or the University of North Texas Health Science Center Institutional Animal Care and Use Committee.

## Supporting information


**Data S1:** micc70034‐sup‐0001‐supinfo.docx.

## Data Availability

Data is available in the University of Michigan's Deep Blue Data Repository (https://doi.org/10.7302/ezyq‐8c12).

## References

[1] S. F. Mohammed , S. Hussain , S. A. Mirzoyev , W. D. Edwards , J. J. Maleszewski , and M. M. Redfield , “Coronary Microvascular Rarefaction and Myocardial Fibrosis in Heart Failure With Preserved Ejection Fraction,” Circulation 131, no. 6 (2015): 550–559. 25552356 10.1161/CIRCULATIONAHA.114.009625 PMC4324362

[2] C. Olianti , I. Costantini , F. Giardini , et al., “3D Imaging and Morphometry of the Heart Capillary System in Spontaneously Hypertensive Rats and Normotensive Controls,” Scientific Reports 10 (2020): 1014276. 10.1038/s41598-020-71174-9 PMC7459314 32868776

[3] L. Gagnon , A. F. Smith , D. A. Boas , A. Devor , T. W. Secomb , and S. Sakadžić , “Modeling of Cerebral Oxygen Transport Based on In Vivo Microscopic Imaging of Microvascular Network Structure, Blood Flow, and Oxygenation,” Frontiers in Computational Neuroscience 10 (2016): 82. 27630556 10.3389/fncom.2016.00082 PMC5006088

[4] D. A. Beard , K. A. Schenkman , and E. O. Feigl , “Myocardial Oxygenation in Isolated Hearts Predicted by an Anatomically Realistic Microvascular Transport Model,” American Journal of Physiology. Heart and Circulatory Physiology 285, no. 5 (2003): H1826–H1836. 12869375 10.1152/ajpheart.00380.2003

[5] P. Fughelli , A. Stella , and A. V. Sterpetti , “Marcello Malpighi (1628–1694),” Circulation Research 124, no. 10 (2019): 1430–1432. 31071004 10.1161/CIRCRESAHA.119.314936

[6] P. Hernández‐Morera , I. Castaño‐González , C. M. Travieso‐González , B. Mompeó‐Corredera , and F. Ortega‐Santana , “Quantification and Statistical Analysis Methods for Vessel Wall Components From Stained Images With Masson's Trichrome,” PLoS One 11, no. 1 (2016): e0146954. 26761643 10.1371/journal.pone.0146954 PMC4711946

[7] S. J. Sangaralingham , E. L. Ritman , P. M. McKie , et al., “Cardiac Micro‐Computed Tomography Imaging of the Aging Coronary Vasculature,” Circulation. Cardiovascular Imaging 5, no. 4 (2012): 518–524. 22679058 10.1161/CIRCIMAGING.112.973057 PMC3408091

[8] H. Razavi , M. N. Dusch , S. Y. Zarafshar , C. A. Taylor , and J. A. Feinstein , “A Method for Quantitative Characterization of Growth in the 3‐D Structure of Rat Pulmonary Arteries,” Microvascular Research 83, no. 2 (2012): 146–153. 22230111 10.1016/j.mvr.2011.12.003

[9] L. Zagorchev , P. Oses , Z. W. Zhuang , et al., “Micro Computed Tomography for Vascular Exploration,” Journal of Angiogenesis Research 2 (2010): 27. 20298533 10.1186/2040-2384-2-7 PMC2841094

[10] C. L. N. Sy , “Integrated Experimental and Modelling Study of Coronary Flow Mechanics,” ResearchSpace@Auckland, 2025, https://hdl.handle.net/2292/64242.

[11] T. Liebmann , N. Renier , K. Bettayeb , P. Greengard , M. Tessier‐Lavigne , and M. Flajolet , “Three‐Dimensional Study of Alzheimer's Disease Hallmarks Using the iDISCO Clearing Method,” Cell Reports 16, no. 4 (2016): 1138–1152. 27425620 10.1016/j.celrep.2016.06.060 PMC5040352

[12] T. Miyawaki , S. Morikawa , E. A. Susaki , et al., “Visualization and Molecular Characterization of Whole‐Brain Vascular Networks With Capillary Resolution,” Nature Communications 11, no. 1 (2020): 1104. 10.1038/s41467-020-14786-z PMC7046771 32107377

[13] J. Grayson , J. W. Davidson , A. Fitzgerald‐Finch , and C. Scott , “The Functional Morphology of the Coronary Microcirculation in the Dog,” Microvascular Research 8, no. 1 (1974): 20–43. 4213442 10.1016/0026-2862(74)90061-2

[14] J. B. Bassingthwaighte , T. Yipintsoi , and R. B. Harvey , “Microvasculature of the Dog Left Ventricular Myocardium,” Microvascular Research 7, no. 2 (1974): 229–249. 4596001 10.1016/0026-2862(74)90008-9 PMC3175795

[15] G. S. Kassab and Y. C. Fung , “Topology and Dimensions of Pig Coronary Capillary Network,” American Journal of Physiology 267, no. 1 Pt 2 (1994): H319–H325. 8048597 10.1152/ajpheart.1994.267.1.H319

[16] X. Ji , T. Ferreira , B. Friedman , et al., “Brain Microvasculature Has a Common Topology With Local Differences in Geometry That Match Metabolic Load,” Neuron 109, no. 7 (2021): 1168–1187. 33657412 10.1016/j.neuron.2021.02.006 PMC8525211

[17] J. Zhu , Y. Deng , T. Yu , X. Liu , D. Li , and D. Zhu , “Optimal Combinations of Fluorescent Vessel Labeling and Tissue Clearing Methods for Three‐Dimensional Visualization of Vasculature,” Neurophotonics 9, no. 4 (2022): 045008. 36466188 10.1117/1.NPh.9.4.045008 PMC9709454

[18] K. H. Dobariya , D. Goyal , and H. Kumar , “Molecular Signature‐Based Labeling Techniques for Vascular Endothelial Cells,” Acta Histochemica 127, no. 1 (2025): 152222. 39644518 10.1016/j.acthis.2024.152222

[19] T. Seidel , J.‐C. Edelmann , and F. B. Sachse , “Analyzing Remodeling of Cardiac Tissue: A Comprehensive Approach Based on Confocal Microscopy and 3D Reconstructions,” Annals of Biomedical Engineering 44, no. 5 (2016): 1436–1448. 26399990 10.1007/s10439-015-1465-6 PMC4805509

[20] J. G. Bensley , R. De Matteo , R. Harding , and M. J. Black , “Three‐Dimensional Direct Measurement of Cardiomyocyte Volume, Nuclearity, and Ploidy in Thick Histological Sections,” Scientific Reports 6, no. 1 (2016): 23756. 27048757 10.1038/srep23756 PMC4822151

[21] G. B. Sands , J. L. Ashton , M. L. Trew , et al., “It's Clearly the Heart! Optical Transparency, Cardiac Tissue Imaging, and Computer Modelling,” Progress in Biophysics and Molecular Biology 168 (2022): 18–32. 34126113 10.1016/j.pbiomolbio.2021.06.005 PMC12076525

[22] I. Nehrhoff , J. Ripoll , R. Samaniego , M. Desco , and M. V. Gómez‐Gaviro , “Looking Inside the Heart: A See‐Through View of the Vascular Tree,” Biomedical Optics Express 8, no. 6 (2017): 3110–3118. 28663930 10.1364/BOE.8.003110 PMC5480453

[23] V. V. Tuchin , I. L. Maksimova , D. A. Zimnyakov , I. L. Kon , A. H. Mavlyutov , and A. A. Mishin , “Light Propagation in Tissues With Controlled Optical Properties,” Journal of Biomedical Optics 2, no. 4 (1997): 401–417. 23014964 10.1117/12.281502

[24] T. Yu , J. Zhu , D. Li , and D. Zhu , “Physical and Chemical Mechanisms of Tissue Optical Clearing,” iScience 24, no. 3 (2021): 102178. 33718830 10.1016/j.isci.2021.102178 PMC7920833

[25] E. A. Susaki , K. Tainaka , D. Perrin , et al., “Whole‐Brain Imaging With Single‐Cell Resolution Using Chemical Cocktails and Computational Analysis,” Cell 157, no. 3 (2014): 726–739. 24746791 10.1016/j.cell.2014.03.042

[26] E. A. Susaki , K. Tainaka , D. Perrin , H. Yukinaga , A. Kuno , and H. R. Ueda , “Advanced CUBIC Protocols for Whole‐Brain and Whole‐Body Clearing and Imaging,” Nature Protocols 10, no. 11 (2015): 1709–1727. 26448360 10.1038/nprot.2015.085

[27] K. Matsumoto , T. T. Mitani , S. A. Horiguchi , et al., “Advanced CUBIC Tissue Clearing for Whole‐Organ Cell Profiling,” Nature Protocols 14, no. 12 (2019): 3506–3537. 31748753 10.1038/s41596-019-0240-9

[28] K. Tainaka , S. I. Kubota , T. Q. Suyama , et al., “Whole‐Body Imaging With Single‐Cell Resolution by Tissue Decolorization,” Cell 159, no. 4 (2014): 911–924. 25417165 10.1016/j.cell.2014.10.034

[29] V. Vigneshwaran , C. L. Sy , B. H. Smaill , G. B. Sands , and N. P. Smith , “Extended‐Volume Image‐Derived Models of Coronary Microcirculation,” Microcirculation 30, no. 5–6 (2023): e12820. 37392132 10.1111/micc.12820

[30] C. Olianti , F. Giardini , E. Lazzeri , et al., “Optical Clearing in Cardiac Imaging: A Comparative Study,” Progress in Biophysics and Molecular Biology 168 (2022): 10–17. 34358555 10.1016/j.pbiomolbio.2021.07.012

[31] H. Corrodi and G. Jonsson , “THE Formaldehyde Fluorescence Method for the Histochemical Demonstration of Biogenic Monoamines A Review on the Methodology,” Journal of Histochemistry and Cytochemistry 15, no. 2 (1967): 65–78.

[32] Z. Zhang , H. Fan , W. Richardson , B. Z. Gao , and T. Ye , “Management of Autofluorescence in Formaldehyde‐Fixed Myocardium: Choosing the Right Treatment,” European Journal of Histochemistry 67, no. 4 (2023): 3812. 37781779 10.4081/ejh.2023.3812 PMC10614721

[33] J. W. Willows , M. Blaszkiewicz , A. Lamore , et al., “Visualization and Analysis of Whole Depot Adipose Tissue Neural Innervation,” IScience 24, no. 10 (2021): 103127. 34622172 10.1016/j.isci.2021.103127 PMC8479257

[34] D. He , T. Li , X. Yang , Y. Xu , and H. Sun , “Sudan Black B Treatment for Reducing Autofluorescence in Human Glioma Tissue and Improving Fluorescent Signals of Bacterial LPS Staining,” Journal of Biophotonics 16, no. 5 (2023): e202200357. 36633394 10.1002/jbio.202200357

[35] J. D. Tune , A. G. Goodwill , H. E. Baker , et al., “Chronic High‐Rate Pacing Induces Heart Failure With Preserved Ejection Fraction‐Like Phenotype in Ossabaw Swine,” Basic Research in Cardiology 117, no. 1 (2022): 50. 36222894 10.1007/s00395-022-00958-z PMC12010922

[36] N. Sakr , O. Glazova , L. Shevkova , et al., “Characterizing and Quenching Autofluorescence in Fixed Mouse Adrenal Cortex Tissue,” International Journal of Molecular Sciences 24, no. 4 (2023): 3432. 36834842 10.3390/ijms24043432 PMC9968082

[37] T. Karpishin , “Reducing Tissue Autofluorescence: Innovations in Immunofluorescent Analysis Boost Signal‐to‐Noise Ratios,” BioTechniques 64, no. 3 (2018): 131.

[38] E. A. Hoffman , B. L. Frey , L. M. Smith , and D. T. Auble , “Formaldehyde Crosslinking: A Tool for the Study of Chromatin Complexes,” Journal of Biological Chemistry 290, no. 44 (2015): 26404–26411. 26354429 10.1074/jbc.R115.651679 PMC4646298

[39] L. Qi , E. K. Knapton , X. Zhang , T. Zhang , C. Gu , and Y. Zhao , “Pre‐Culture Sudan Black B Treatment Suppresses Autofluorescence Signals Emitted From Polymer Tissue Scaffolds,” Scientific Reports 7, no. 1 (2017): 8361. 28827657 10.1038/s41598-017-08723-2 PMC5567053

[40] O. N. Shilova , E. S. Shilov , and S. M. Deyev , “The Effect of Trypan Blue Treatment on Autofluorescence of Fixed Cells,” Cytometry. Part A 91, no. 9 (2017): 917–925. 10.1002/cyto.a.23199 28857464

[41] “Nikon Microscopy Resolution Calculator,” 2025, https://www.microscope.healthcare.nikon.com/microtools/resolution‐calculator/.

[42] S. van der Walt , J. L. Schönberger , J. Nunez‐Iglesias , et al., “Scikit‐Image: Image Processing in Python,” PeerJ 2 (2014): 2e453. 10.7717/peerj.453 PMC4081273 25024921

[43] W. Niblack , An Introduction to Digital Image Processing (Prentice‐Hall International, 1986).

[44] R. Verweji , “nd2reader: A Pure‐Python Package for Reading Nikon. nd2 Files,” 2025, https://open‐science‐tools.github.io/nd2reader/index.html#.

[45] Dask Development Team , “Dask: Library for Dynamic Task Scheduling.”

[46] “3D Slicer Image Computing Platform [3D Slicer],” 2025, https://slicer.org/.

[47] R. Kikinis , S. D. Pieper , and K. G. Vosburgh , “3D Slicer: A Platform for Subject‐Specific Image Analysis, Visualization, and Clinical Support,” in Intraoperative Imaging and Image‐Guided Therapy, ed. F. A. Jolesz (Springer, 2024), 277–289.

[48] A. Fedorov , R. Beichel , J. Kalpathy‐Cramer , et al., “3D Slicer as an Image Computing Platform for the Quantitative Imaging Network,” Magnetic Resonance Imaging 30, no. 9 (2012): 1323–1341. 22770690 10.1016/j.mri.2012.05.001 PMC3466397

[49] N. Kaneko , R. Matsuda , M. Toda , and K. Shimamoto , “Three‐Dimensional Reconstruction of the Human Capillary Network and the Intramyocardial Micronecrosis,” American Journal of Physiology. Heart and Circulatory Physiology 300, no. 3 (2011): H754–H761. 21148764 10.1152/ajpheart.00486.2010

[50] B. G. Anderson and W. D. Anderson , “Microvasculature of the Canine Heart Demonstrated by Scanning Electron Microscopy,” American Journal of Anatomy 158, no. 2 (1980): 217–227. 7416057 10.1002/aja.1001580210

[51] R. Battistella , M. Kritsilis , H. Matuskova , et al., “Not All Lectins Are Equally Suitable for Labeling Rodent Vasculature,” International Journal of Molecular Sciences 22, no. 21 (2021): 11554. 34768985 10.3390/ijms222111554 PMC8584019

[52] R. T. Robertson , S. T. Levine , S. M. Haynes , et al., “Use of Labeled Tomato Lectin for Imaging Vasculature Structures,” Histochemistry and Cell Biology 143, no. 2 (2015): 225–234. 25534591 10.1007/s00418-014-1301-3

[53] “Invitrogen *Lycopersicon Esculentum* (Tomato) Lectin (LEL, TL), DyLight 594 1 mg | Invitrogen & Trade; | Fisher Scientific,” 2025, https://www.fishersci.com/shop/products/lycopersicon‐esculentum‐tomato‐lectin‐lel‐tl‐dylight‐594/L32471.

[54] “DyLight 594 NHS Ester 1 mg | Thermo Scientific | Thermofisher.com,” 2025, https://www.thermofisher.com/order/catalog/product/46412.

[55] “P01870 IGHG_RABIT UniProt. [P01870 IGHG_RABIT UniProt],” 2025, https://www.uniprot.org/uniprotkb/P01870/entry.

[56] “A0A1Y1BBR2 A0A1Y1BBR2_RABIT UniProt [A0A1Y1BBR2 A0A1Y1BBR2_RABIT UniProt],” 2025, https://www.uniprot.org/uniprotkb/A0A1Y1BBR2/entry#names_and_taxonomy.

[57] “Anti‐Mouse IgG (H+L), Highly Cross Adsorbed Antibody Produced in Donkey Affinity Isolated Antibody, Buffered Aqueous Solution | Sigma‐Aldrich,” 2025, https://www.sigmaaldrich.com/US/en/product/sigma/sab3701102#product‐documentation.

[58] H. Kolesová , M. Čapek , B. Radochová , J. Janáček , and D. Sedmera , “Comparison of Different Tissue Clearing Methods and 3D Imaging Techniques for Visualization of GFP‐Expressing Mouse Embryos and Embryonic Hearts,” Histochemistry and Cell Biology 146, no. 2 (2016): 141–152. 27145961 10.1007/s00418-016-1441-8

[59] I. Nehrhoff , D. Bocancea , J. Vaquero , et al., “3D Imaging in CUBIC‐Cleared Mouse Heart Tissue: Going Deeper,” Biomedical Optics Express 7, no. 9 (2016): 3716–3720. 27699132 10.1364/BOE.7.003716 PMC5030044

[60] F. Perbellini , A. K. L. Liu , S. A. Watson , I. Bardi , S. M. Rothery , and C. M. Terracciano , “Free‐Of‐Acrylamide SDS‐Based Tissue Clearing (FASTClear) for Three Dimensional Visualization of Myocardial Tissue,” Scientific Reports 7, no. 1 (2017): 5188. 28701763 10.1038/s41598-017-05406-w PMC5507863

[61] H. R. Ueda , A. Ertürk , K. Chung , et al., “Tissue Clearing and Its Applications in Neuroscience,” Nature Reviews. Neuroscience 21, no. 2 (2020): 61–79. 31896771 10.1038/s41583-019-0250-1 PMC8121164

[62] A. S. Davis , A. Richter , S. Becker , et al., “Characterizing and Diminishing Autofluorescence in Formalin‐Fixed Paraffin‐Embedded Human Respiratory Tissue,” Journal of Histochemistry and Cytochemistry 62, no. 6 (2014): 405–423. 24722432 10.1369/0022155414531549 PMC4174629

[63] M. Casper , H. Schulz‐Hildebrandt , M. Evers , R. Birngruber , D. Manstein , and G. Hüttmann , “Optimization‐Based Vessel Segmentation Pipeline for Robust Quantification of Capillary Networks in Skin With Optical Coherence Tomography Angiography,” Journal of Biomedical Optics 24, no. 4 (2019): 046005. 31041858 10.1117/1.JBO.24.4.046005 PMC6990060

[64] A. P. Di Giovanna , A. Tibo , L. Silvestri , et al., “Whole‐Brain Vasculature Reconstruction at the Single Capillary Level,” Scientific Reports 8, no. 1 (2018): 12573. 30135559 10.1038/s41598-018-30533-3 PMC6105658

[65] D. Halpern and T. W. Secomb , “The Squeezing of Red Blood Cells Through Capillaries With Near‐Minimal Diameters,” Journal of Fluid Mechanics 203 (1989): 381–400.

[66] S. P. Bishop and J. L. Drummond , “Surface Morphology and Cell Size Measurement of Isolated Rat Cardiac Myocytes,” Journal of Molecular and Cellular Cardiology 11, no. 5 (1979): 423–433. 448740 10.1016/0022-2828(79)90467-x

[67] F. R. Heinzel , V. Bito , P. G. A. Volders , G. Antoons , K. Mubagwa , and K. R. Sipido , “Spatial and Temporal Inhomogeneities During Ca^2+^ Release From the Sarcoplasmic Reticulum in Pig Ventricular Myocytes,” Circulation Research 91, no. 11 (2002): 1023–1030. 12456488 10.1161/01.res.0000045940.67060.dd

[68] M. Taper , G. Carrington , M. Peckham , S. Lal , and R. D. Hume , “A Comparison of Fixation and Immunofluorescence Protocols for Successful Reproducibility and Improved Signal in Human Left Ventricle Cardiac Tissue,” Journal of Microscopy 296, no. 1 (2024): 34–47. 38856969 10.1111/jmi.13336

