# Supplementary material for: Immersion‐Based Clearing and Autofluorescence Quenching in Myocardial Tissue
Source: Microcirculation. 2025 Nov 5;32(8):e70034. doi: 10.1111/micc.70034 (PMC12589860; doi:10.1111/micc.70034)
Supplement: Supplementary file 1 — Data S1: micc70034‐sup‐0001‐supinfo.docx. [file MICC-32-e70034-s001.docx]

Supplemental Material for

“Immersion-based Clearing and Autofluorescence Quenching in Myocardial Tissue”

**
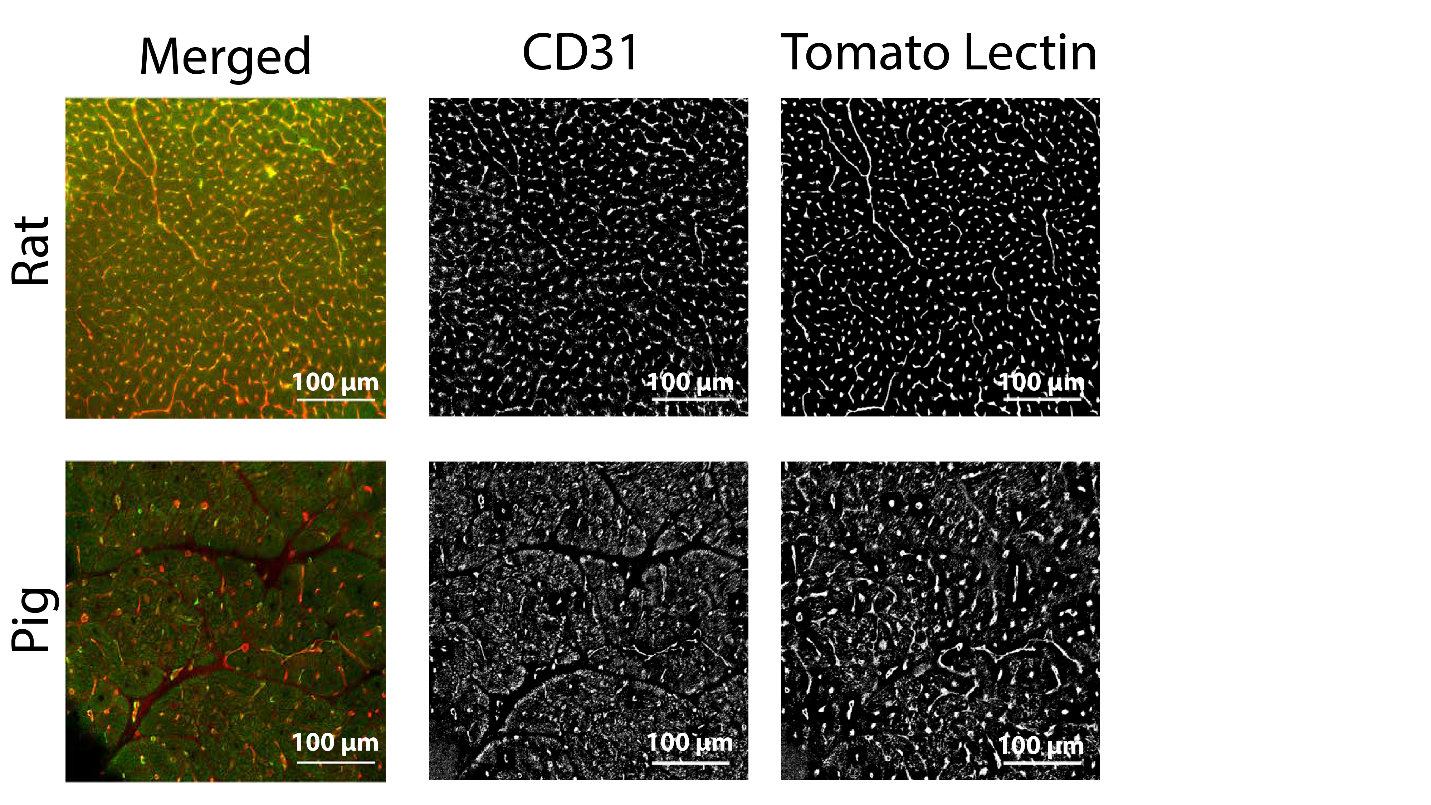
**

Supplemental Figure 1: Merged images of CD31 and tomato lectin (left) with binary masks of CD31 (middle) and tomato lectin (right) for both the rat myocardial sample (top row) and the pig myocardial sample (bottom row).


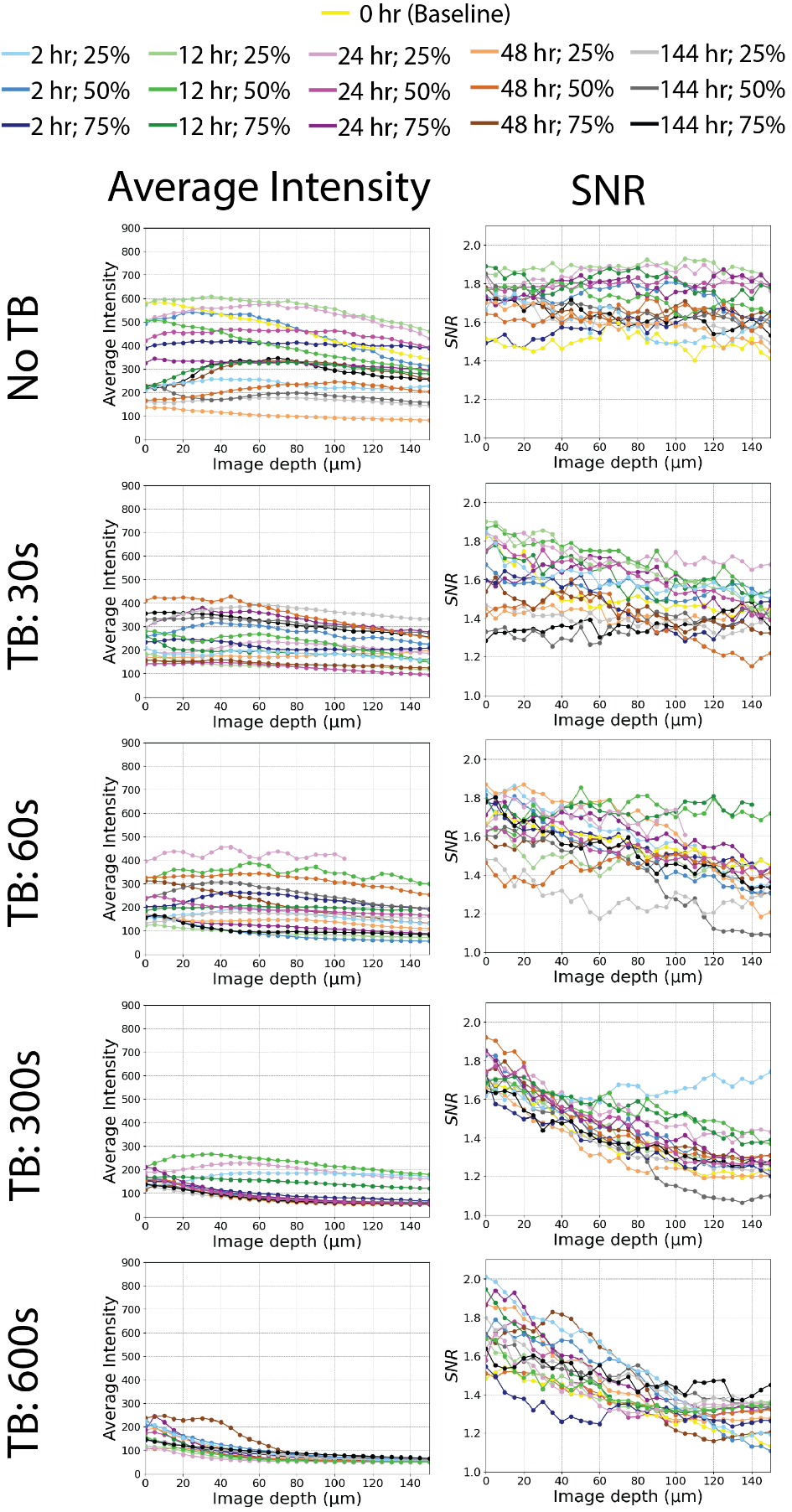


Supplemental Figure 2: Intensity (left) and SNR (right) versus image depth for rat myocardial tissue sections with varying Reagent I incubation times (1 hour, 6 hours, 12 hours, 24 hours and 72 hours) and diluted Reagent I concentrations (25%, 50%, and 75%). Each row presents results for Various TrueBlack® (TB) incubation times (0, 30, 60, 300, and 600 seconds).


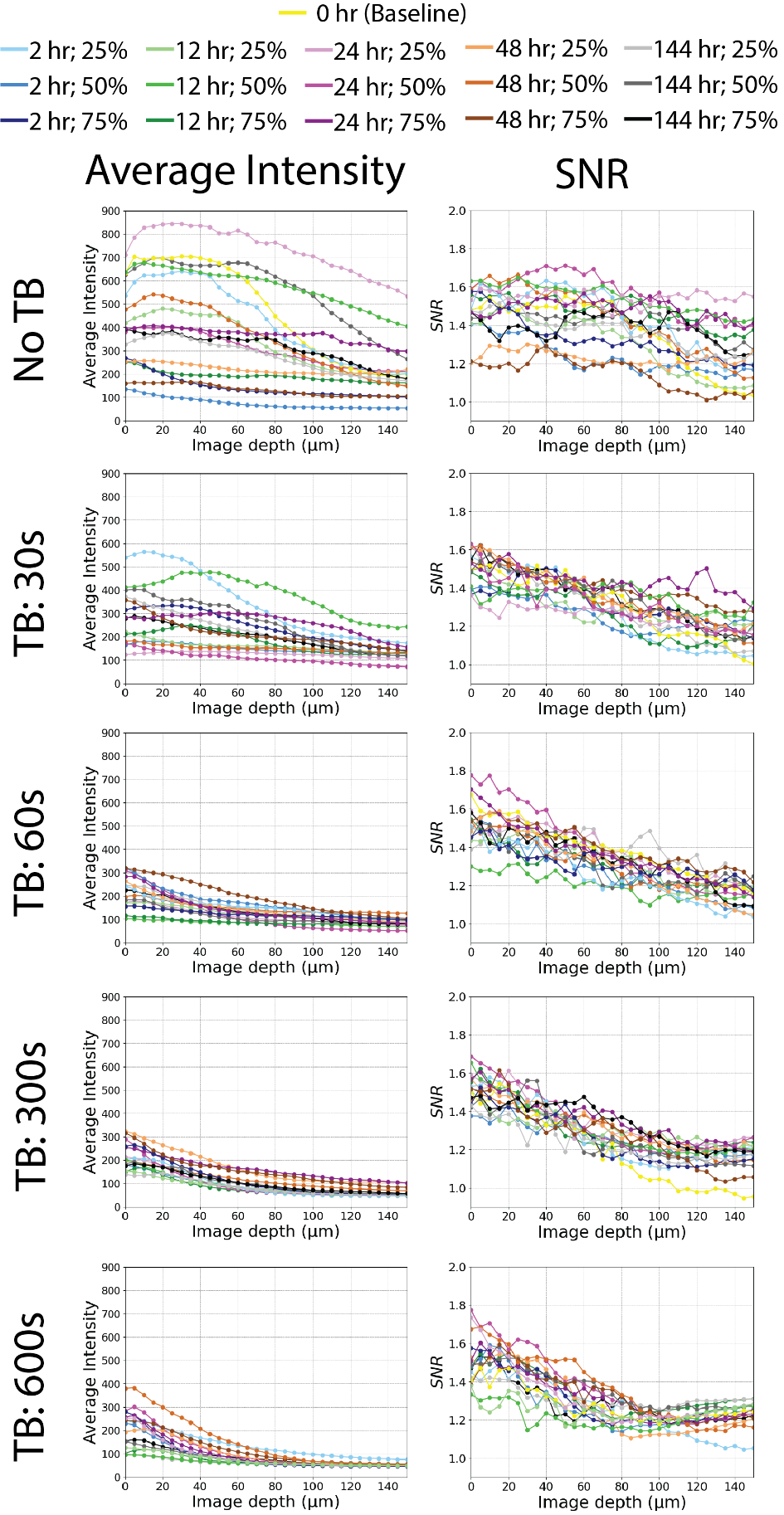


Supplemental Figure 3: Intensity (left) and SNR (right) versus image depth for pig myocardial tissue sections with varying Reagent I incubation times (1 hour, 6 hours, 12 hours, 24 hours and 72 hours) and diluted Reagent I concentrations (25%, 50%, and 75%). Each row presents results for Various TrueBlack® (TB) incubation times (0, 30, 60, 300, and 600 seconds).


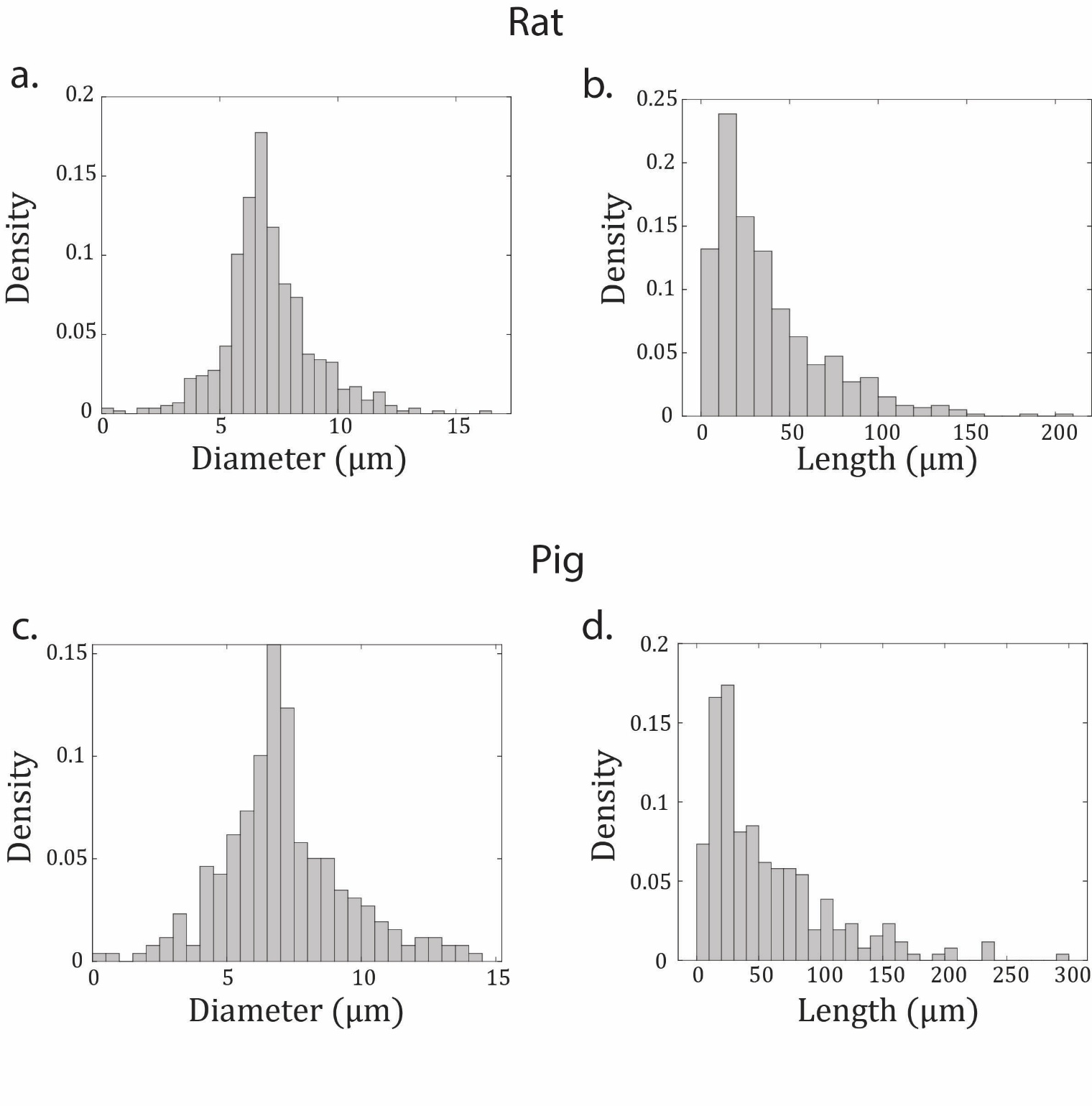


Supplemental Figure 4: Histograms of capillary diameters and lengths form rat (a and b) and pig (c and d) capillary networks shown in Figure 10.


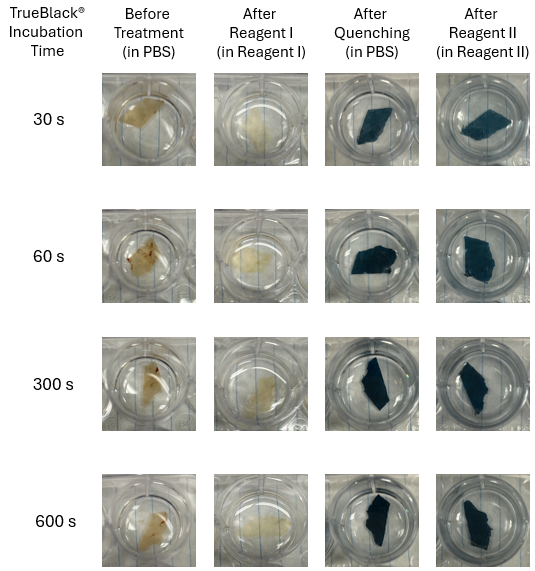


Supplemental Figure 5: Changes in sample coloration after each step of the clearing protocol for pig myocardial samples treated with TrueBlack® for varying incubation times


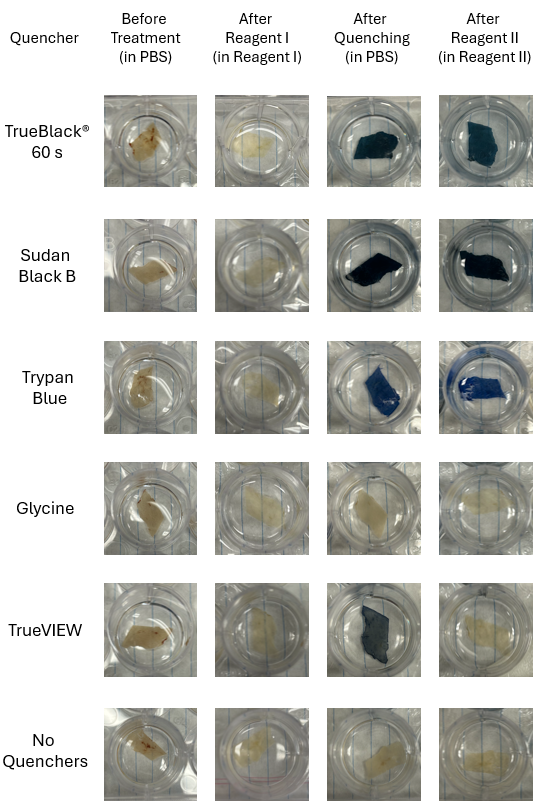


Supplemental Figure 6: Changes in sample coloration after each step of the clearing protocol for pig myocardial samples treated with different quenchers

Supplemental Table 1: Results of Anova for rat delipidation experiment at Z=0 μm

|  | Degrees of freedom | SSE | MSE | F-value | P-value |
| --- | --- | --- | --- | --- | --- |
| Reagent I Concentration | 2 | 0.00545 | 0.00273 | 0.378 | 0.697 |
| Reagent I Time | 4 | 0.0766 | 0.0191 | 2.67 | 0.112 |
| Residuals | 8 | 0.0577 | 0.00721 |  |  |

Supplemental Table 2: Results of Tukey HSD test for rat delipidation experiment; Z=0 μm

| Comparison of Reagent I Concentration | | | Comparison of Reagent I Concentration | | |
| --- | --- | --- | --- | --- | --- |
| Comparison | Difference | Adjusted  p-value | Comparison | Difference | Adjusted  p-value |
| 25% vs 50% | 0.0109 | 0.977 | 2 vs 12 hrs | -0.205 | 0.0981 |
| 25% vs 75% | 0.0448 | 0.694 | 2 vs 24 hrs | -0.152 | 0.272 |
| 50% vs 75% | 0.0339 | 0.808 | 2 vs 48 hrs | -0.076 | 0.802 |
|  |  |  | 2 vs 144 hrs | -0.151 | 0.277 |
|  |  |  | 12 vs 24 hrs | 0.053 | 0.933 |
|  |  |  | 12 vs 48 hrs | 0.129 | 0.406 |
|  |  |  | 12 vs 144 hrs | 0.054 | 0.929 |
|  |  |  | 24 vs 48 hrs | 0.076 | 0.807 |
|  |  |  | 24 vs 144 hrs | 0.001 | 1.00 |
|  |  |  | 48 vs 144 hrs | -0.075 | 0.814 |

Supplemental Table 3: Results of Anova for rat delipidation experiment at Z=150 μm

|  | Degrees of freedom | SSE | MSE | F-value | P-value |
| --- | --- | --- | --- | --- | --- |
| Reagent I Concentration | 2 | 0.00676 | 0.00338 | 0.767 | 0.496 |
| Reagent I Time | 4 | 0.130 | 0.0324 | 7.35 | 0.00868 |
| Residuals | 8 | 0.0353 | 0.00441 |  |  |

Supplemental Table 4: Results of Tukey HSD test for rat delipidation experiment; Z=150 μm

| Comparison of Reagent I Concentration | | | Comparison of Reagent I Concentration | | |
| --- | --- | --- | --- | --- | --- |
| Comparison | Difference | Adjusted  p-value | Comparison | Difference | Adjusted  p-value |
| 25% vs 50% | -0.0219 | 0.863 | 2 vs 12 hrs | -0.103 | 0.386 |
| 25% vs 75% | -0.0518 | 0.468 | 2 vs 24 hrs | -0.149 | 0.132 |
| 50% vs 75% | -0.02984 | 0.764 | 2 vs 48 hrs | 0.0575 | 0.821 |
|  |  |  | 2 vs 144 hrs | 0.0953 | 0.454 |
|  |  |  | 12 vs 24 hrs | -0.0455 | 0.911 |
|  |  |  | 12 vs 48 hrs | 0.161 | 0.0977 |
|  |  |  | 12 vs 144 hrs | 0.199 | **0.0378** |
|  |  |  | 24 vs 48 hrs | 0.206 | **0.0312** |
|  |  |  | 24 vs 144 hrs | 0.244 | **0.0126** |
|  |  |  | 48 vs 144 hrs | 0.038 | 0.951 |

Supplemental Table 5: Results of Anova for pig delipidation experiment at Z=0 μm

|  | Degrees of freedom | SSE | MSE | F-value | P-value |
| --- | --- | --- | --- | --- | --- |
| Reagent I Concentration | 2 | 0.0311 | 0.0156 | 1.11 | 0.377 |
| Reagent I Time | 4 | 0.0797 | 0.0199 | 1.42 | 0.312 |
| Residuals | 8 | 0.113 | 0.0141 |  |  |

Supplemental Table 6: Results of Tukey HSD test for pig delipidation experiment; Z=0 μm

| Comparison of Reagent I Concentration | | | Comparison of Reagent I Concentration | | |
| --- | --- | --- | --- | --- | --- |
| Comparison | Difference | Adjusted  p-value | Comparison | Difference | Adjusted  p-value |
| 25% vs 50% | -0.110 | 0.357 | 2 vs 12 hrs | -0.0463 | 0.987 |
| 25% vs 75% | -0.0374 | 0.874 | 2 vs 24 hrs | -0.0412 | 0.992 |
| 50% vs 75% | 0.0723 | 0.618 | 2 vs 48 hrs | 0.154 | 0.541 |
|  |  |  | 2 vs 144 hrs | 0.0280 | 0.998 |
|  |  |  | 12 vs 24 hrs | 0.00510 | 1.000 |
|  |  |  | 12 vs 48 hrs | 0.200 | 0.318 |
|  |  |  | 12 vs 144 hrs | 0.0743 | 0.933 |
|  |  |  | 24 vs 48 hrs | 0.195 | 0.339 |
|  |  |  | 24 vs 144 hrs | 0.0692 | 0.947 |
|  |  |  | 48 vs 144 hrs | -0.126 | 0.698 |

Supplemental Table 7: Results of Anova for pig delipidation experiment at Z=150 μm

|  | Degrees of freedom | SSE | MSE | F-value | P-value |
| --- | --- | --- | --- | --- | --- |
| Reagent I Concentration | 2 | 0.00123 | 0.000617 | 0.0468 | 0.954 |
| Reagent I Time | 4 | 0.170 | 0.0424 | 3.22 | 0.0749 |
| Residuals | 8 | 0.105 | 0.0132 |  |  |

Supplemental Table 8: Results of Tukey HSD test for pig delipidation experiment; Z=150 μm

| Comparison of Reagent I Concentration | | | Comparison of Reagent I Concentration | | |
| --- | --- | --- | --- | --- | --- |
| Comparison | Difference | Adjusted  p-value | Comparison | Difference | Adjusted  p-value |
| 25% vs 50% | -0.00813 | 0.993 | 2 vs 12 hrs | -0.112 | 0.754 |
| 25% vs 75% | 0.0138 | 0.980 | 2 vs 24 hrs | -0.268 | 0.112 |
| 50% vs 75% | 0.0220 | 0.951 | 2 vs 48 hrs | 0.0367 | 0.994 |
|  |  |  | 2 vs 144 hrs | -0.0738 | 0.927 |
|  |  |  | 12 vs 24 hrs | -0.156 | 0.500 |
|  |  |  | 12 vs 48 hrs | 0.1488 | 0.542 |
|  |  |  | 12 vs 144 hrs | 0.0383 | 0.993 |
|  |  |  | 24 vs 48 hrs | 0.305 | 0.066 |
|  |  |  | 24 vs 144 hrs | 0.195 | 0.315 |
|  |  |  | 48 vs 144 hrs | -0.115 | 0.763 |

Supplemental Table 9: Results of ANOVA for rat TrueBlack® experiment

| ANOVA for Percent Relative Intensity | | | | | | | | | | |
| --- | --- | --- | --- | --- | --- | --- | --- | --- | --- | --- |
|  | Degrees of freedom | | SSE | | MSE | | F-value | | P-value | |
| TrueBlack® Incubation Time | 4 | | 44146 | | 11036 | | 20.6 | | 3.00E-11 | |
| Residuals | 70 | | 37523 | | 536 | |  | |  | |
| ANOVA for SNR Z=0 μm | | | | | | | | | | |
|  | | Degrees of freedom | | SSE | | MSE | | F-value | | P-value |
| TrueBlack® Incubation Time | | 4 | | 0.157 | | 0.0392 | | 2.044 | | 0.0976 |
| Residuals | | 70 | | 1.34 | | 0.0191 | |  | |  |
| ANOVA for SNR Z=105 μm | | | | | | | | | | |
|  | | Degrees of freedom | | SSE | | MSE | | F-value | | P-value |
| TrueBlack® Incubation Time | | 4 | | 1.35 | | 0.338 | | 23.4 | | 2.7 E-12 |
| Residuals | | 70 | | 1.01 | | 0.0145 | |  | |  |

Supplemental Table 10: Results of Tukey HSD for rat TrueBlack® experiment

|  | Percent Relative Intensity | | SNR  Z=0 μm | | | SNR  Z=105 μm | |
| --- | --- | --- | --- | --- | --- | --- | --- |
| Comparison | Difference | Adjusted  p-value | | Difference | Adjusted  p-value | Difference | Adjusted  p-value |
| 0 vs 30 s | 0.144 | 0.441 | | 0.126 | 0.105 | **0.228** | **1.84 E-5** |
| 0 vs 60 s | 0.199 | 0.141 | | 0.0607 | 0.999 | **0.194** | **3.31 E-4** |
| 0 vs 300 s | 0.462 | **6.51 E-6** | | 0.0131 | 0.751 | **0.340** | **<1E-6** |
| 0 vs 600 s | 0.6788 | **<1E-6** | | 0.0199 | 0.995 | **0.384** | **<1E-6** |
| 30 vs 60s | 0.0553 | 0.965 | | -0.0652 | 0.699 | -0.0341 | 0.936 |
| 30 vs 300 s | 0.318 | **0.00306** | | -0.0113 | 0.181 | 0.112 | 0.0890 |
| 30 vs 600 s | 0.535 | **<1E-6** | | -0.106 | 0.234 | **0.157** | **0.00619** |
| 60 vs 300 s | 0.263 | **0.0218** | | -0.0475 | 0.880 | **0.147** | **0.0114** |
| 60 vs 600 s | 0.480 | **2.85 E-6** | | -0.0408 | 0.928 | **0.190** | **4.72 E-4** |
| 300 vs 600 s | 0.217 | 0.0888 | | 0.00678 | 1.000 | 0.0431 | 0.863 |

Supplemental Table 11: Results of ANOVA for pig TrueBlack® experiment

| ANOVA for Percent Relative Intensity | | | | | | | | | | |
| --- | --- | --- | --- | --- | --- | --- | --- | --- | --- | --- |
|  | Degrees of freedom | | SSE | | MSE | | F-value | | P-value | |
| TrueBlack® Incubation Time | 4 | | 16049 | | 4012 | | 17.55 | | 5.14 E-10 | |
| Residuals | 70 | | 16005 | | 229 | |  | |  | |
| ANOVA for SNR Z=0 μm | | | | | | | | | | |
|  | | Degrees of freedom | | SSE | | MSE | | F-value | | P-value |
| TrueBlack® Incubation Time | | 4 | | 0.0266 | | 0.00665 | | 0.526 | | 0.717 |
| Residuals | | 70 | | 0.884 | | 0.0126 | |  | |  |
| ANOVA for SNR Z=105 μm | | | | | | | | | | |
|  | | Degrees of freedom | | SSE | | MSE | | F-value | | P-value |
| TrueBlack® Incubation Time | | 4 | | 0.235 | | 0.0587 | | 7.18 | | 6.60 E-5 |
| Residuals | | 70 | | 0.572 | | 0.00817 | |  | |  |

Supplemental Table 12: Results of Tukey HSD for pig TrueBlack® experiment

|  | Percent Relative Intensity | | SNR  Z=0 μm | | | SNR  Z=105 μm | |
| --- | --- | --- | --- | --- | --- | --- | --- |
| Comparison | Difference | Adjusted  p-value | | Difference | Adjusted  p-value | Difference | Adjusted  p-value |
| 0 vs 30 s | 0.0469 | 0.914 | | -0.0422 | 0.842 | 0.0883 | 0.0679 |
| 0 vs 60 s | 0.164 | **0.0322** | | -0.0503 | 0.736 | **0.115** | **0.00769** |
| 0 vs 300 s | 0.342 | **< 1E -6** | | -0.0498 | 0.743 | **0.155** | **1.22 E-4** |
| 0 vs 600 s | 0.355 | **< 1E -6** | | -0.0429 | 0.833 | **0.148** | **2.67 E-4** |
| 30 vs 60s | 0.117 | 0.223 | | -0.00811 | 1.00 | 0.0262 | 0.931 |
| 30 vs 300 s | 0.295 | **1.07 E -5** | | -0.00768 | 1.00 | 0.0666 | 0.268 |
| 30 vs 600 s | 0.308 | **4.27 E-6** | | 0.00078 | 1.00 | 0.0595 | 0.379 |
| 60 vs 300 s | 0.178 | **0.0163** | | 0.00043 | 1.00 | 0.0404 | 0.738 |
| 60 vs 600 s | 0.191 | **0.00816** | | 0.00733 | 1.00 | 0.0333 | 0.850 |
| 300 vs 600 s | 0.0130 | 0.999 | | 0.00690 | 1.00 | -0.00706 | 1.000 |

Supplemental Table 13: Results of ANOVA for rat quencher experiment

| ANOVA for Percent Relative Intensity | | | | | | | | | | |
| --- | --- | --- | --- | --- | --- | --- | --- | --- | --- | --- |
|  | Degrees of freedom | | SSE | | MSE | | F-value | | P-value | |
| Quencher | 5 | | 5230 | | 1046 | | 21.4 | | 9.23 E-4 | |
| Residuals | 6 | | 293 | | 48.8 | |  | |  | |
| ANOVA for SNR Z=0 μm | | | | | | | | | | |
|  | | Degrees of freedom | | SSE | | MSE | | F-value | | P-value |
| Quencher | | 5 | | 0.0176 | | 0.00352 | | 0.244 | | 0.928 |
| Residuals | | 6 | | 0.0865 | | 0.0144 | |  | |  |
| ANOVA for SNR Z=105 μm | | | | | | | | | | |
|  | | Degrees of freedom | | SSE | | MSE | | F-value | | P-value |
| Quencher | | 5 | | 0.465 | | 0.0930 | | 14.86 | | 0.00251 |
| Residuals | | 6 | | 0.0376 | | 0.00626 | |  | |  |

Supplemental Table 14: Results of Tukey HSD for rat quencher experiment

|  | Percent Relative Intensity | | SNR Z=0 μm | | SNR Z=150 μm | |
| --- | --- | --- | --- | --- | --- | --- |
| Comparison | Difference | Adjusted  p-value | Difference | Adjusted  p-value | Difference | Adjusted  p-value |
| No Quencher (-Q) vs TrueBlack® | -3.78 | 0.992 | -0.0563 | 0.996 | **0.459** | **0.00867** |
| No Quencher (-Q) vs  Sudan Black B | **32.3** | **0.0260** | -0.0359 | 0.999 | 0.283 | 0.0775 |
| No Quencher (-Q) vs Trypan Blue | 21.2 | 0.141 | 0.0628 | 0.993 | 0.138 | 0.551 |
| No Quencher (-Q) vs TrueVIEW | -3.38 | 0.995 | -0.0278 | 1.000 | -0.0783 | 0.906 |
| No Quencher (-Q) vs Glycine | **-33.6** | **0.0217** | -0.0299 | 1.000 | -0.0727 | 0.928 |
| TrueBlack® vs Sudan Black B | **36.0** | **0.0155** | 0.0204 | 1.000 | -0.176 | 0.344 |
| TrueBlack® vs Trypan Blue | 25.0 | 0.0774 | 0.119 | 0.905 | **-0.321** | **0.0463** |
| TrueBlack® vs TrueVIEW | 0.401 | 1.00 | 0.0285 | 1.000 | **-0.537** | **0.00385** |
| TrueBlack® vs Glycine | **-29.8** | **0.0373** | 0.0264 | 1.000 | **-0.532** | **0.00407** |
| Sudan Black B vs Trypan Blue | -11.1 | 0.635 | 0.0986 | 0.953 | -0.144 | 0.514 |
| Sudan Black B vs TrueVIEW | **-35.6** | **0.0163** | 0.00808 | 1.000 | **-0.361** | **0.0275** |
| Sudan Black B vs Glycine | **-65.8** | **6.49 E-4** | 0.00595 | 1.000 | **-0.356** | **0.0295** |
| Trypan Blue vs TrueVIEW | -24.6 | 0.0824 | -0.0906 | 0.966 | -0.217 | 0.197 |
| Trypan Blue vs Glycine | **-54.8** | **0.00179** | -0.0927 | 0.963 | -0.211 | 0.213 |
| Glycine vs  TrueVIEW | **-30.2** | **0.0352** | -0.00213 | 1.000 | 0.00555 | 1.000 |

Supplemental Table 15: Results of ANOVA for pig quencher experiment

| ANOVA for Percent Relative Intensity | | | | | | | | | | |
| --- | --- | --- | --- | --- | --- | --- | --- | --- | --- | --- |
|  | Degrees of freedom | | SSE | | MSE | | F-value | | P-value | |
| Quencher | 5 | | 5079 | | 1015.8 | | 21.75 | | 8.83 E-4 | |
| Residuals | 6 | | 280 | | 46.7 | |  | |  | |
| ANOVA for SNR Z=0 μm | | | | | | | | | | |
|  | | Degrees of freedom | | SSE | | MSE | | F-value | | P-value |
| Quencher | | 5 | | 0.0825 | | 0.0165 | | 4.21 | | 0.0545 |
| Residuals | | 6 | | 0.0235 | | 0.00391 | |  | |  |
| ANOVA for SNR Z=105 μm | | | | | | | | | | |
|  | | Degrees of freedom | | SSE | | MSE | | F-value | | P-value |
| Quencher | | 5 | | 0.0521 | | 0.0104 | | 3.246 | | 0.0921 |
| Residuals | | 6 | | 0.0193 | | 0.00321 | |  | |  |

Supplemental Table 16: Results of Tukey HSD for pig quencher experiment

|  | Percent Relative Intensity | | SNR Z=0 μm | | SNR Z=150 μm | |
| --- | --- | --- | --- | --- | --- | --- |
| Comparison | Difference | Adjusted  p-value | Difference | Adjusted  p-value | Difference | Adjusted  p-value |
| No Quencher (-Q) vs TrueBlack® | **51.5** | **0.00222** | -0.118 | 0.482 | 0.0213 | 0.998 |
| No Quencher (-Q) vs  Sudan Black B | **65.7** | **5.76 E-4** | -0.198 | 0.121 | 0.144 | 0.242 |
| No Quencher (-Q) vs Trypan Blue | **31.3** | **0.0271** | 0.0381 | 0.986 | 0.106 | 0.490 |
| No Quencher (-Q) vs TrueVIEW | 26.7 | 0.0539 | 0.0125 | 1.000 | -0.0480 | 0.947 |
| No Quencher (-Q) vs Glycine | **30.6** | **0.0297** | -0.0770 | 0.809 | 0.00775 | 1.000 |
| TrueBlack® vs Sudan Black B | 14.3 | 0.397 | -0.0798 | 0.789 | 0.123 | 0.363 |
| TrueBlack® vs Trypan Blue | -20.2 | 0.154 | 0.157 | 0.255 | 0.0850 | 0.677 |
| TrueBlack® vs TrueVIEW | -24.7 | 0.0736 | 0.131 | 0.396 | -0.069 | 0.813 |
| TrueBlack® vs Glycine | -20.8 | 0.139 | 0.0414 | 0.980 | -0.0135 | 1.000 |
| Sudan Black B vs Trypan Blue | **-34.5** | **0.0172** | 0.236 | 0.0621 | -0.0382 | 0.979 |
| Sudan Black B vs TrueVIEW | **-39.0** | **0.00938** | 0.211 | 0.0972 | -0.193 | 0.0939 |
| Sudan Black B vs Glycine | **-35.1** | **0.0158** | 0.121 | 0.462 | -0.137 | 0.281 |
| Trypan Blue vs TrueVIEW | -4.54 | 0.980 | -0.0257 | 0.998 | -0.154 | 0.200 |
| Trypan Blue vs Glycine | -0.624 | 1.00 | -0.115 | 0.507 | -0.0985 | 0.556 |
| Glycine vs TrueVIEW | 3.92 | 0.989 | -0.0895 | 0.713 | 0.0558 | 0.908 |
